# Which Psychological Factors are Related to HIV Testing? A Quantitative Systematic Review of Global Studies

**DOI:** 10.1007/s10461-015-1246-0

**Published:** 2015-11-13

**Authors:** Michael Evangeli, Kirsten Pady, Abigail L. Wroe

**Affiliations:** Department of Psychology, Royal Holloway University of London, Egham, Surrey TW20 0EX UK

**Keywords:** HIV, Testing, Psychosocial, Psychological, Systematic review

## Abstract

Deciding to test for HIV is necessary for receiving HIV treatment and care among those who are HIV-positive. This article presents a systematic review of quantitative studies on relationships between psychological (cognitive and affective) variables and HIV testing. Sixty two studies were included (fifty six cross sectional). Most measured lifetime testing. HIV knowledge, risk perception and stigma were the most commonly measured psychological variables. Meta-analysis was carried out on the relationships between HIV knowledge and testing, and HIV risk perception and testing. Both relationships were positive and significant, representing small effects (HIV knowledge, *d* = 0.22, 95 % CI 0.14–0.31, *p* < 0.001; HIV risk perception, OR 1.47, 95 % CI 1.26–1.67, *p* < 0.001). Other variables with a majority of studies showing a relationship with HIV testing included: perceived testing benefits, testing fear, perceived behavioural control/self-efficacy, knowledge of testing sites, prejudiced attitudes towards people living with HIV, and knowing someone with HIV. Research and practice implications are outlined.

## Introduction

HIV testing is a prerequisite for receiving HIV treatment and care among those who are HIV-positive. Early diagnosis and access to treatment is associated with a reduced likelihood of onward transmission [1], better response to antiretroviral treatment (ART), and reduced mortality and morbidity [2]. However, many people living with HIV are unaware of their status. The World Health Organisation (WHO) estimates that less than half of those infected with HIV have been diagnosed [3]. The growing availability of ART reinforces the need to scale up testing interventions. To develop interventions that are effective in increasing uptake, it is crucial to study the factors that may influence the decision to test [4].

Current WHO recommendations state that all HIV testing should be informed, voluntary and confidential [5]. Historically, voluntary counselling and testing (VCT) has been the dominant model, with individuals actively seeking an HIV test from a healthcare or community facility [6]. Client or self-initiated testing has been the main focus of increasing access initiatives, including through the use of mobile VCT centres [7] and home-based counselling and testing [8], which address testing barriers such as travel costs and confidentiality concerns [9].

Greater provider-initiated, routine, testing was recommended by the WHO in 2007 as an additional strategy to increase testing uptake [10]. This involves healthcare providers offering HIV testing to individuals attending facilities as a standard component of medical care (e.g., antenatal care), with the individual actively ‘opting out’ if they do not want to be tested [10]. However, while it is recommended that testing be routinely offered to groups with specific risk factors (e.g., in sexual health clinics in all contexts), it is not cost-effective to offer testing to all individuals presenting to health services unless in generalised epidemic settings [10]. Indeed, the WHO recommends a strategic mix of different models of testing delivered by a range of providers, including lay providers [5]. There remain, therefore, a significant proportion of the HIV positive population whose diagnosis is still reliant on uptake of VCT. Recent self-testing initiatives have further highlighted the importance of individual psychological factors related to HIV testing decision-making [11].

Social cognition models, including the Theory of Planned Behavior (TPB) [12] and the Health Belief Model (HBM) [13], have highlighted the importance of individual proximal determinants of health behaviour. Many of these models, including the TPB, suggest that the likelihood of performing a given behaviour is dependent on the strength of intention to perform the behaviour, which, in turn, is influenced by other psychological factors (such as behavioural attitudes) [12]. For example, with reference to the TPB, behavioural HIV testing attitudes might include beliefs about the benefits of testing (e.g., “*HIV testing helps people to access medication if they are HIV*-*positive*) or the cons of testing (e.g., *“HIV testing is not confidential”*). The relationship between psychological factors and testing is potentially moderated by non-psychological factors, such as testing context (i.e., client vs. provider-initiated), regional resource availability and the nature of the population. For example, it may be that differing levels of HIV risk perception between MSM and heterosexual populations are important in explaining differences in HIV testing uptake [14]. Researching demographic and structural associations with testing is necessary for targeting interventions to appropriate populations [15, 16]. It is, however, also crucial to understand psychological factors that are associated with the decision to test or not to test for HIV, as these factors are likely to mediate the relationships between higher level factors (interpersonal and extrapersonal) and testing, are more proximal to testing decision-making and are potentially modifiable. This review focuses on associations between psychological factors and HIV testing.

Previous reviews of psychological associations with HIV testing have often focused on resource-rich contexts. In one review [17], studies were limited to high-income countries, with 34/50 (68 %) studies from the U.S.A. A second review [18] only included studies conducted in Europe. Grey literature (unpublished literature including dissertations and conference abstracts) was omitted from both of these reviews. A third recent review focused on intrapersonal, interpersonal and extrapersonal barriers to testing in Australia, Canada and the UK [19]. These reviews are helpful in starting to understand psychological factors that are associated with the decision to opt for or against HIV testing, and they highlighted important issues in relation to testing such as the fear of death and personal risk perception [18]. It is not possible, however, to conclude that correlates of testing will be similar in resource rich and resource limited contexts. For example, the nature of the relationship between HIV risk perception and HIV testing may be different in contexts where there are differing levels of accessibility to HIV care and treatment. This review, therefore, has no regional restrictions. This study also fills an important gap in the literature by conducting meta-analyses of the statistical relationships between psychological factors and HIV testing where there are enough studies to support this approach. This has not been conducted in other reviews [17, 18, 20]. Inclusion of meta-analyses means that the magnitude of effects can be evaluated [21]. In comparison with previous reviews [17–19], this article focuses only on studies that assess the quantitative relationships between psychological variables and testing (rather than combining quantitative and qualitative findings) to facilitate assessment of the strength of relationships with HIV testing. The main objective of this review, therefore, is to critically analyse and synthesise data from a comprehensive range of studies investigating the quantitative relationship between psychological (cognitive and affective) variables and HIV testing.

## Method

### Study Eligibility Criteria

This study followed PRISMA Statement guidelines [22] for the reporting of systematic reviews. Studies were included if they:

1. Used a quantitative design;
2. Included participants who had the capacity to make a decision to test for HIV. Studies of populations requiring parental/guardian consent to undergo an HIV test (e.g. children under the age of 15 or with profound learning disabilities), or for whom HIV testing was mandatory (e.g. some state prisoners in the U.S.A.) were excluded. The target population of this review was therefore predominantly individuals aged ≥15 years;
3. Measured psychological variables. Studies that focused explicitly only on psychological *responses* to HIV testing (such as measuring mood directly after testing) were excluded. ‘Psychological’ variables were more specifically defined as cognitive and affective variables, relating to an individual’s internal state (e.g. feelings or beliefs); and
4. Measured whether an HIV test was taken or not, according to self-report or patient records. Because it was considered unlikely that most studies would specify the mode of testing, for example, whether it was client-initiated (where the focus of the decision was whether to opt for or against testing), or provider-initiated (where the decision was whether to accept an offer or opt out of testing), all modes of testing were included.

### Sources of Information

Studies published in peer-reviewed journals were retrieved from the electronic databases Pubmed/Medline, PsycINFO, Web of Science and the Cochrane Library. In addition, the search included papers from conference proceedings (International AIDS conference, AIDS Impact, International AIDS Society Conference), and the Networked Digital Library of Theses and Dissertations (NDLTD). The search was restricted to studies conducted since January 1, 1996. This was due to biomedical advances in 1996, which led to the uptake of effective antiretroviral regimens that have greatly improved the health and life expectancy of people with HIV/AIDS. Any studies using data collected before this date were excluded.

### Search Strategy

The searches were conducted using combinations of the following terms: ‘HIV testing’, ‘psychological’, ‘psychosocial’, ‘psychiatric’, ‘cognitive’, ‘affective’, ‘behavioural (behavioral)’, ‘psychopathology’, ‘mood’, ‘beliefs’, ‘illness perception’ and ‘illness representation’. ‘HIV testing’ was searched for as a keyword in the title, whereas the psychological terms were searched for as keywords in the title or abstract.

### Data Collection

**Fig. 1:**
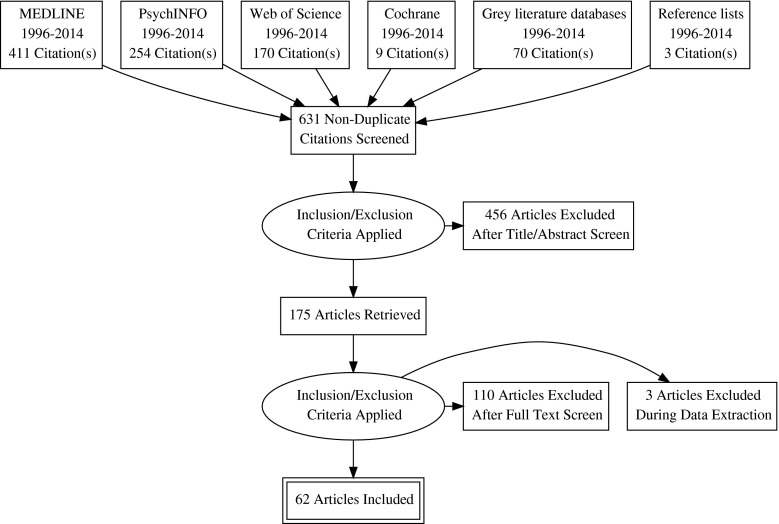
Study Search Process

Following recommendations of PRISMA [22], the data collection process had four stages (see Fig. 1). One reviewer (KP) carried out the searches for the identification of studies, using pre-specified search criteria. This was completed on 1st October 2014. All duplications were removed. Two reviewers (KP and one of two undergraduate reviewers) independently screened the remaining titles and abstracts for eligibility. Articles considered relevant by either reviewer were retrieved in full text. The two reviewers then independently assessed eligibility of the retrieved articles. Exclusions were reported, with reasons given. Any disagreements were resolved by a third reviewer (ME or AW), to result in a final group of studies for analysis.

### Data Abstraction and Quality Assessment

The following details were extracted from the articles (by KP, verified by ME): authors, date of publication, location, design, nature of sample, age, gender, ethnicity, definition and measurement of psychological and testing variables, context and measurement of testing behaviour, and nature of relationship between psychological variables and testing. Methodological quality was assessed, using criteria adapted from Siegfried et al. [23]. Articles were assessed on two dimensions of external validity (sample representativeness and response rate) and four dimensions of internal validity (performance bias, detection bias, attrition bias and selection bias/confounding) (see Table 1).

**Table 1:** Methodological quality assessment

*External validity*	
Representativeness of sample	• Was the sample representative of the target population (consecutive or random sample) or were all of the population eligible?
Percentage of selected individuals whom agreed to participate	• Were at least 80 % of those eligible to participate in all groups (where relevant) recruited?
*Internal validity*	
Performance bias	• Was there an objective method for measuring whether HIV testing took place? Was there at least one non self- report measure, e.g. clinic records?
Detection bias	• Were measures of psychological variables objective or of established reliability and validity?
Attrition bias	• Were at least 80 % of those invited to participate in the study included in final analysis (for intervention/cohort studies)?
Selection bias/control of possible confounding variables	• Were possible confounding variables (a) measured (b) considered in the analysis?

KP and one undergraduate reviewer assessed all articles independently, before comparing ratings. Any disagreements were resolved through discussion between KP and the undergraduate reviewer. Ongoing disagreement was resolved by ME or AW.

### Statistical Analysis

Inter-rater reliability for study eligibility was assessed using Cohen’s Kappa. Meta-analyses were conducted on the associations between selected psychological variables and HIV testing. A minimum of 15 studies measuring the association between a specific variable and testing was required for eligibility for meta-analysis, based on evidence of bias with the use of meta-analysis with small numbers of studies [24]. Effect sizes (either standardised mean differences [*d*] or odds ratios [OR]) were calculated for the relationship with testing for each study sample. The use of either *d* or OR in each meta-analysis was determined by data provided by the majority of studies (e.g., the majority of studies measuring HIV knowledge used *d*, hence the few studies that that used OR were converted to *d* [25]). If any data were missing, authors were contacted to supply the information. R-3.1.2 (http://www.r-project.org/) was used to conduct the meta-analyses and assess heterogeneity, outliers and influence, and publication bias. Random effects models were used as there could be no assumption the samples were drawn from a homogenous population. Further permutation tests were run due to the small number of studies included in each meta-analysis [26]. Cochran’s *Q* test (testing differences between study effect sizes) and the *I*^2^ test [27] (measuring the extent of inconsistency among study effect sizes) were used to test for heterogeneity between studies (with *I*^2^ values 25, 50 and 75 % equivalent to low, moderate and high levels of inconsistency [28]).

Publication bias was assessed using both Rosenberg’s Fail-Safe N [29] and the trim and fill method [30]. Rosenberg’s Fail-Safe N estimates the number of non-significant unpublished studies required to eliminate a significant overall effect size. Fail-Safe numbers do not take into account sample size or variance of the studies, however. Therefore, the trim and fill method was also used. This method tests and adjusts for funnel plot asymmetry that may be caused by studies with small samples showing small effects being less likely to be published than similar sized studies showing larger effects.

## Results

### Study Characteristics

The search identified 62 studies eligible for inclusion (see Fig. 1).

There was strong agreement between the reviewers on eligibility (Cohen’s Kappa = 0.85, *p* < 0.001). Thirty-six articles were published between January 2010 and October 2014 [31–66], and 24 were published in 2000–2009 [67–90]. Only two articles published prior to 2000 were included [91, 92]. Twenty articles described research conducted in sub-Saharan Africa [34, 36–38, 47–51, 54, 58, 59, 62, 64, 67, 76, 77, 81, 85, 90]. Another 21 were conducted in North America [32, 35, 40, 42, 45, 55, 57, 61, 66, 68, 69, 71, 73, 74, 78, 80, 84, 86, 87, 91, 92], one in South America [93], and four in the Caribbean [33, 82, 83, 89]. Nine were conducted in Asia [39, 43, 44, 46, 52, 63, 65, 79, 88], five in Europe [41, 53, 60, 70, 72], and one in Australia [31]. One study [75] incorporated findings from both sub-Saharan African and Asian regions.

The majority of studies (n = 56) were cross-sectional (measuring both psychological factors and testing at the same time point) [31–44, 46, 47, 49–53, 56, 57, 59–70, 72–88, 90–92]. Forty-nine of the cross sectional studies asked about historical HIV testing (e.g., any lifetime testing) [31, 34–44, 46, 49–53, 56, 57, 59–70, 72, 73, 75–80, 82–84, 86–88, 90, 91], and seven measured whether testing was undertaken at the time of study [32, 33, 47, 74, 81, 85, 92]. There were four prospective cohort studies [45, 55, 58, 71], one case–control study [54], and one intervention study [48].

Testing context (client or provider-initiated) was not generally specified, with the exception of a few studies which restricted the outcome variable to VCT [43, 54, 88, 91]. One study [62] provided data for several testing outcomes, including client and provider-initiated testing. Prospective studies gave more detail on testing context. Two studies [58, 85] reported acceptance of antenatal testing, and three [33, 47, 92] specified ‘voluntary’ testing at the clinic or study site. A summary of the 62 selected studies is presented in Table 2.

**Table 2:** Study proforma

References	Location, inclusion/exclusion and testing context	Design and sampling	Measurement of psychological variables	Measure of testing behaviour	Associations between psychological variables and testing
Adam et al. [31]	Australia Urban and rural areas MSM aged ≥16 years	Cross-sectional Convenience sampling Response rate 73.7 % *n* = 309 Mean age 29.3 years	*HIV-related knowledge*: 8 items. Dichotomous response options *Perceived susceptibility to HIV*: 2 items. Likert scale, *α* = 0.91 *Perceived severity of HIV* 1 item. Likert scale *Perceived pros of testing*: 10 items. Likert scale, *α* = 0.83 *Perceived cons of testing* 11 items. Likert scale, *α* = 0.81 *Positive vs. negative attitudes to testing*: 5 item. Likert scale, *α* = 0.91. *Subjective norms*: 5 items. Likert scale, *α* = 0.92 *Perceived behavioural control* 5 items. Likert scale, *α* = 0.91 *Fear of testing*: 11 items. Likert scale, *α* = 0.89 *Perceived stigma* 15 items. Likert scale, *α* = 0.85 Scales developed for current study	Self-reported previous HIV testing: Not tested/non routine testing/ Moderate routine testing/ Highly routine testing	*HIV-related knowledge:* significantly associated with testing routine (univariate *p* < 0.001; multivariate *p* < 0.05) *Perceived susceptibility to HIV*, *Ns* *Perceived severity of HIV*, *Ns* *Perceived pros of testing*: Significantly associated with testing routine (univariate *p* < 0.001; multivariate, *p* < 0.001). Moderate-routine, no-routine and non-testers perceived less pros than highly routine testers (AOR 0.20, *p* < 0.001; AOR 0.15, *p* < 0.001; AOR 0.09, *p* < 0.001, respectively) *Perceived cons of testing*: significantly associated with testing routine in univariate (*p* < 0.001) but not multivariate analysis (*ns*) *Positive vs. negative attitudes to testing*: significantly associated with testing routine (univariate, *p* < 0.001; multivariate, *p* < 0.01). Moderate-routine, no-routine and non-testers perceived less positives of testing than highly-routine testers (AOR 0.54, *p* < 0.05; AOR 0.36, *p* < 0.001; AOR 0.36, *p* < 0.01, respectively) *Subjective norms*: significantly associated with testing routine in univariate (*p* < 0.001) but not multivariate analysis (*ns*) *Perceived behavioural control*: significantly associated with testing routine (univariate, *p* < 0.001; multivariate, *p* < 0.001). Moderate-routine, no-routine and non-testers perceived less behavioural control than highly-routine testers (AOR 0.32, *p* < 0.05; AOR 0.27, *p* < 0.01; AOR 0.16, *p* < 0.001, respectively) *Fear of testing:* significantly associated with testing routine in univariate (*p* < 0.001) but not multivariate analysis (*ns*) *Perceived stigma:* significantly associated with testing routine in univariate (*p* < 0.001) but not multivariate analysis (*ns*). No-routine testers perceived more stigma than highly-routine testers (AOR 1.91, *p* < 0.05)
Andrinopoulos et al. [33]	Jamaica HIV-negative Male inmates of correctional facility aged ≥18 years Voluntary testing while incarcerated	Cross-sectional Stratified random sampling by facility section Response rate 89 % *n* = 298 Age range 18–68 years	*HIV coping self-efficacy*: 7 items. Likert, Adapted from [129–131], *α* 0.86 *External stigma*: 9 items. Likert scale. Adapted from [132, 133], *α* = 0.83 *Internal stigma*: 4 items. Likert scale. Adapted from [134], *α* = 0.84 *HIV testing stigma*: 6 items. Likert scale. Adapted from [135], *α* = 0.74 *Perceived current risk of HIV infection*: 1 item. Likert scale *Perceived social support*: 17 items. Likert. Adapted [136], *α* 0.92 *HIV-related knowledge*: 13 items. Dichotomous response options. Adapted from [132, 137], *α* = 0.68	Accepting HIV test	*HIV coping self-efficacy*: high coping self-efficacy associated with higher likelihood of testing (OR 2.05, 1.43–2.93, *p* < 0.001; AOR 1.86, 1.24–2.78, *p* = 0.003) *External stigma*: *Ns* (OR 1.03, 0.67–1.59, *p* = 0.90) *Internal stigma*: *Ns* (OR 1.09, 0.84–1.41, *p* = 0.51) *HIV testing stigma:* low testing stigma associated with higher likelihood of testing (OR 1.69, 1.17–2.44, *p* = 0.01; AOR 1.71, 1.05–2.79, *p* = 0.03) *Perceived current risk of HIV infection*: Perceiving risk associated with higher likelihood of testing (OR 1.94, 1.27–2.97, *p* = 0.002; AOR 2.51, 1.57–4.01, *p* < 0.001) *Perceived social support*: *Ns* (OR 1.11, 0.83–1.49, *p* = 0.47) *HIV-related knowledge*: *Ns* (OR 1.21, 0.92–1.60, *p* = 0.18)
Berendes and Rimal [34]	Malawi Urban areas Adolescents and adults resident in selected townships	Cross-sectional Systematic random sampling *n* = 890. 407 (45.7 %) males, 483 females (54.3 %). Age range 12–88 years	*HIV-related knowledge*: 12 items. Dichotomous response options, *α* = 0.59 *Self-efficacy*: 8 items. Likert scale, *α* = 0.90 *Stigma towards people living with HIV (PLWH):* 9 items. Dichotomous response options, *α* = 0.65 All developed for current study	Self-reported previous HIV testing	*HIV-related knowledge* Positive association with previous testing (*B* = 0.03, 0.01–0.05, *p* < 0.05) *Self-efficacy* Positive association with previous testing (*B* = 0.47, 0.16–0.78, *p* < 0.01) *Stigma towards PLWHA* Negative association with HIV testing (*B* = −0.85, −1.64 to −0.06, *p* < 0.05)
Berkley-Patton et al. [35]	U.S.A. Urban areas African American, church-affiliated	Cross-sectional Convenience sampling. *n* = 210: 77 (36.6 %) males, 133 (63.3 %) females. 18–87 years. 188 (89.4 %) African Americans, 22 (10.6 %) other ethnicity	*HIV-related knowledge:* 10 items. Dichotomous response options. From [138], *α* = 0.66 *Intention to test annually for HIV*: 3 items. Likert scale. From [139], *α* = 0.91	Self-reported previous HIV testing: Lifetime and Last 12 months	*HIV-related knowledge* Significant association with lifetime HIV testing in univariate (*r* = 0.19, *p* < 0.01) but not multivariate analysis (AOR 1.05, 0.83–1.33, *ns*) *Intention to test annually for HIV* Significant association with lifetime testing in univariate (*r* = 0.17, *p* < 0.05) but not multivariate analysis (AOR 1.03, 0.94–1.13, *ns*) Significant association with testing in last 12 months (*r* = 0.33, *p* < 0.01; AOR 1.21, 1.08–1.35, *p* < 0.01)
Bogart et al. [67]	South Africa HIV-negative individuals using STI clinics	Cross–sectional Convenience sampling, *n* = 783 471 (60.2 %) males, 312 (39.8 %) females. Mean age = 28.9 years. 736 (94 %) Black African, 47 (6 %) other ethnicity	*HIV-related knowledge* 11 items with dichotomous response options. Adapted from [140], *α* = 0.71 *HIV-related stigma* 11 items on Likert scale From [141], *α* = 0.71 *Belief in AIDS-related genocidal conspiracy* 1 item developed for current study *Knowing someone* *with HIV/AIDS*	Self-reported previous HIV testing	*HIV-related knowledge* *Ns* (AOR 1.06, 0.98–1.14). 1.*HIV-related stigma* *Ns* (AOR 0.82, 0.60–1.10) *Belief in AIDS-related genocidal conspiracy* Belief in genocidal conspiracy significantly associated with less testing (AOR 0.85, 0.74–0.98, *p* < 0.05) *Knowing someone with HIV/AIDS* *Ns* (AOR 1.23, 0.89–1.69)
Bohnert and Latkin [68]	U.S.A. Urban. Aged ≥18 years. African Americans. High drug use rate. No recent enrolment in HIV behavioural intervention	Cross-sectional Respondent-driven sampling *n* = 1430 880 (61.5 %) males, 551 (38.5 %) females	*Belief in AIDS-related conspiracy theories* 2 items on Likert scale Developed for current study *Depression* 20 items on Likert scale From [142] *α* = 0.90	Self-reported previous HIV testing	*Belief in AIDS-related conspiracy theories* Individuals with conspiracy beliefs less likely to have never tested (OR 0.51, 0.28–0.92, *p* < 0.05; AOR 0.43, 1.30–4.30, *p* < 0.01) *Depression* Individuals with depression more likely to have never tested (OR 1.38, 0.90–2.12, *ns*; AOR 1.61, 1.02–2.52, *p* < 0.05)

References	Location, inclusion/exclusion and testing context	Design and sampling	Measurement of psychological variables	Measure of testing behaviour	Associations between psychological variables and testing
Burchell et al. [69]	Canada Testing while incarcerated Adult inmates of correctional centres (serving <2 years)	Cross-sectional Stratified sampling by correctional centre. Response rate 89 %, *n* = 597 439 (73.5 %) males, 158 (26.5 %) females Age 18–40+ years	*Perceived future risk for HIV infection (while incarcerated)* 1 item on Likert scale *Attitude towards* *mandatory HIV testing policy* 1 item on Likert scale Developed for current study *Knowing someone with HIV/AIDS inside prison*	Self-reported HIV testing in last year while incarcerated	*Perceived future risk for HIV infection (while incarcerated)* *Ns* (AOR 2.20, 0.98–4.90, *p* = 0.06) *Attitudes towards mandatory HIV testing policy* Individuals who agreed with mandatory testing for correctional staff and inmates more likely to have tested (AOR 2.00, 1.20–3.30, *p* = 0.01) *Knowing someone with HIV/AIDS inside prison* Among 18–29 year olds, significantly associated with testing (AOR 2.70, 1.30–5.70, *p* = 0.01). Among 30–39 year olds, significantly associated with testing (AOR 2.90, 1.30–6.60, *p* = 0.01). Among >40 year olds, *ns* (AOR 0.23, CI 0.05–1.10, *p* = 0.06)
Corno and de Walque [36]	Lesotho. Urban and rural areas. Women aged 15–49, men aged 15–59.	Cross-sectional Stratified sampling by district. *n* = 20,833, 6114 (29.3 %) males, 14,719 (70.7 %) females	*Stigmatising attitudes to PLWHA* 5 items on Likert scale Developed for current study *α* = 0.79 Data from 2004/2009 Demographic and Health Survey (LDHS)	Self-reported previous HIV testing	*Stigmatising attitudes to PLWHA* Negative association between stigmatising attitudes and testing for women (β = −0.03, SE = 0.004, *p* < 0.01; βadj = −0.03, SE = 0.01, *p* < 0.01) and men (β = −0.04, SE = 0.01, *p* < 0.01; βadj = −0.02, SE = 0.01, *p* < 0.01)

References	Location, inclusion/exclusion and testing context	Design and sampling	Measurement of psychological variables	Measure of testing behaviour	Associations between psychological variables and testing
***Creel and Rimal [37]	Namibia Rural and urban areas Sexually active individuals ≥15 years old	Cross-sectional Systematic random sampling *n* = 2671 1211 (45.3 %) males, 1459 (54.7 %) females	*Perceived susceptibility* 1 item on Likert scale *Self-efficacy* 4 items on Likert scale *α* = 0.73	Self-reported previous HIV testing	*Perceived susceptibility* Higher perceived susceptibility associated with less likelihood of testing (AOR 0.89, 0.82–0.97, *p* < 0.01) *Self-efficacy* Higher self-efficacy associated with greater likelihood of testing (AOR 1.24, 1.04–1.48, *p* < 0.05)
Cremin et al. [38]	13 countries in Sub-Saharan Africa. Women aged 15–49, men 15–59 Permanent residents of selected households	Cross-sectional Cluster sampling Response rate, 81.9–98.1 %. *n* = 134,965. 65,867 (48.8 %) males, 69,098 (51.2 %) females	*Stigmatising attitudes to HIV* 1 item on Likert scale	Self-reported previous HIV testing and collection of results	*Stigmatising attitudes to HIV* HIV testing lower among those expressing stigmatising attitudes: in women in Rwanda (AOR 0.75, 0.60–0.93) Ns relationship between stigmatising attitudes and testing in HIV: in women in Zimbabwe (AOR 1.13, 0.91–1.41) and Senegal (AOR 0.60, 0.34–1.06) and in men in Rwanda (AOR 0.90, 0.70–1.16), Zimbabwe (AOR 0.96 (0.72–1.29) and Senegal (AOR 0.55, 0.21–1.41)
Das et al. [39]	India. Urban and rural areas Married men aged 15–54.	Cross-sectional Cluster sampling. *n* = 39257. 87 % response. 21386 (54.5 %) aged 36–54 years	*Knowledge about HIV routes of transmission and prevention* *Knowledge about HIV risk behaviours and prevention*	Self-reported previous HIV testing	*Knowledge about HIV routes of transmission and prevention* Significant association with testing (AOR 1.18, 1.12–1.23, *p* < 0.01) *Knowledge about HIV risk behaviours and prevention* *Ns* association with testing (AOR 1.03, 1.00–1.07)

References	Location, inclusion/exclusion and testing context	Design and sampling	Measurement of psychological variables	Measure of testing behaviour	Associations between psychological variables and testing
Delva et al. [70]	Bosnia and Herzegovina, Macedonia, Serbia and Montenegro. Urban. High school students	Cross-sectional Cluster sampling *n* = 2150. 1022 (47.5 %) males, 1128 (52.5 %) females. Age range 12–24 years (*M* = 16.7 years)	*Self-assessed health* 1 item on Likert scale *Suspicion of having had an STI* 1 item with dichotomous response options *Knows friend or relative with HIV* 1 item with dichotomous response options	Self-reported previous HIV testing	*Self-assessed health* Individuals who self-assessed health as ‘poor/very poor’ more likely to have tested (AOR 6.59, 1.45–29.84, *p* = 0.01) *Suspicion of having had an STI* Individuals who did not suspect they had a previous STI less likely to have tested (AOR 0.29, 0.11–0.79, *p* = 0.01) *Knows friend or relative with HIV* Knowing a friend/relative with HIV associated with testing (AOR 8.67, 3.77–19.95, *p* < 0.0001)
Desai and Rosenheck [71]	18 sites across 9 states, U.S.A. Homeless adults with serious mental illness. Not involved in another treatment program	Prospective cohort. Recruited through outreach services. *n* = 5890. 3599 61.1 %) males, 2289 (38.9 %) females. Mean age = 38.7 years. 2482 (42.2 %) White, 3401 (57.8 %) other ethnicity. 14.8 % attrition.	*Worry about getting AIDS* 1 item on Likert scale	Getting tested for HIV in 3-month follow-up period after contact with programme	*Worry about getting AIDS* Extent of worry positively associated with HIV testing (β = 0.06, SE = 0.03, AOR 1.06, *p* < 0.04)
Dorr et al. [92]	U.S.A. Voluntary HIV testing at student clinic Heterosexual university students	Cross-sectional Convenience sampling. *n* = 111 42 (38 %) males, 69 (62 %) females Mean age 20.3 years. 103 (93 %) White/European, 5 (4.5 %) Asian American, 1 (1 %) African American, 2 (1.5 %) other ethnicity	*Health Belief Model* *Perceived susceptibility* 1 item ‘likelihood of testing positive for HIV in lifetime’ on Likert scale *Perceived severity* 1 item on Likert scale *Perceived benefits* 1 item on Likert scale *Perceived barriers* 1 item on Likert scale *Perceived norms* 1 item on Likert scale Developed for current study *Consideration of Future Consequences (CFC)*. Individuals with higher CFC more influenced by long-term consequences of behaviour, from [143]. 12 items on Likert scale. *α* = 0.84	Undertaking HIV test the same day at the student clinic (comparison group: never having an HIV test)	*Health Belief Model* *Perceived susceptibility* *Ns* (AOR = 0.01, *p* = 0.99) *Perceived severity* Ns (AOR = 0.96, *p* = 0.93) *Perceived benefits* Greater perceived benefits positively associated with testing (AOR = 0.38, *p* < 0.01) *Perceived barriers* *Ns* (AOR 1.61, *p* = 0.08) Perceived norms *Ns* (AOR 0.72, *p* = 0.26) *CFC* Greater CFC positively associated with testing (AOR 0.23, *p* < 0.01)

References	Location, inclusion/exclusion and testing context	Design and sampling	Measurement of psychological variables	Measure of testing behaviour	Associations between psychological variables and testing
Earnshaw et al. [40]	U.S.A. Injecting drug users (IDU) receiving methadone maintenance therapy at clinic	Cross-sectional Convenience sampling Response rate 30.6 %. *n* = 93 47 (50.5 %) males, 46 (49.5 %) females. Mean age 37.1 years. 63 (67.7 %) White, 13 Black (14 %), 13 Latino (14 %), 4 (4.3 %) other ethnicity	**HIV stigma mechanisms** **Stereotypes** *α* = 0.76 *Prejudice* *α* = 0.81 *Discrimination* *α* = 0.73 From [101] *Perceived future risk of HIV* 1 item on Likert scale	Frequency of HIV testing	*HIV stigma mechanisms* *Stereotypes* *Ns* (B = 0.08, SE = 0.21, β = 0.05) *Perceived future risk of HIV* Individuals perceiving lower risk engaged in less frequent testing (B = 0.45, SE = 0.20, β = 0.26, *p* < 0.05)
Fenton et al. [72]	UK. Urban. Migrant Africans.	Cross-sectional Convenience sampling. 74.8 % response rate. *n* = 748. 396 (52.9 %) males, 352 (47.1 %) females Age range 16–70 years. From: Congo/Zaire: 176 (23.5 %), Kenya: 121 (16.2 %), Uganda: 132 (17.6 %), UK: 10 (1.3 %), Zambia: 106 (14.2 %), Zimbabwe: 158 (21.1 %), Other: 45 (6 %)	*Perceived future risk of HIV* *Perceived group norms of using condoms with new partners*	Self-reported previous HIV testing	*Perceived future risk of HIV* Perceived risk positively associated with testing among men (OR 2.35, 1.47–3.76; AOR 2.28, 1.34–3.90) but not women (OR 1.02, 0.63–1.66) *Perceived group norms of using condoms with new partners* Lower perceived group norms ns associated with testing among men (OR 0.78, 0.47–1.30) and women (OR 0.58, 0.31–1.07)
Flowers et al. [41]	United Kingdom Urban area. MSM. Not known to be HIV positive Attending commercial gay venues	Cross-sectional Stratified sampling by time and location Response rates 78 % (year 2000), 62 % (year 2010) *n* = 1382	*Perceived benefits of HV testing* 3 items on Likert scale *Fear of a positive HIV test result* 5 items on Likert scale *Clinic-related barriers* 4 items on Likert scale *Attitudes towards sex with HIV-positive partners* 3 items on Likert scale *Norm for HIV testing* 1 item on Likert scale	Self-reported previous HIV testing: Recent testing (in 12 months prior to survey) Non-recent testing (>12 months prior to survey) Never testing	*Perceived benefits of HV testing* Non-testers perceived less benefits of testing than recent testers (AOR 0.75, 0.60–0.93, *p* = 0.01). *Ns* difference between non-testers and non-recent testers (AOR 0.92, 0.73–1.16, *p* = 0.46). *Ns* difference between non-recent testers and recent testers (AOR 0.82, 0.65–1.02, *p* = 0.07) *Fear of a positive HIV test result* Non-testers had greater fear of a positive result than recent testers (AOR 2.19, 1.76–2.71, *p* < 0.001). Non-testers had greater fear than non-recent testers (AOR 1.53, 1.22–1.93, *p* < 0.001). Non-recent testers had greater fear than recent testers (AOR 1.42, 1.14–1.78, *p* = 0.002) *Clinic-related barriers* *Ns* difference between non-testers and recent testers (AOR 1.19, 0.93–1.51, *p* = 0.17). *Ns* difference between non-testers and non-recent testers (AOR 1.20, 0.92–1.56, *p* = 0.17). *Ns* difference between non-recent testers and recent testers (AOR 0.99, 0.77–1.26, *p* = 0.92) *Attitudes towards sex with HIV-positive partners* Non-testers had more negative attitudes than recent testers (AOR 1.24, 1.04–1.48, *p* = 0.02). Non-testers had more negative attitudes than non-recent testers (AOR 1.35, 1.11–1.63, *p* = 0.002). *Ns* difference between non-recent testers and recent testers (AOR 0.92, 0.78–1.08, *p* = 0.33) *Norm for HIV testing* Non-testers perceived testing to be less of norm than recent testers (AOR 0.57, 0.48–0.67, *p* < 0.001). Non-testers perceived testing to be less of norm than non-recent testers (AOR 0.64, 0.53–0.77, *p* < 0.001). *Ns* difference: non-recent vs. recent testers (AOR 0.89, 0.76–1.05, *p* = 0.16)
Ford et al. [73]	U.S.A. Urban area. Adults ≥18 years attending STI clinic. Black ethnicity Seeking STI diagnosis or screening for possible STI	Cross-sectional Convenience sampling Response rate 87 %. *n* = 408 Age range 18–59 years. 408 (100 %) Black/African American	*Perceived risk of HIV* 1 item From [144] *HIV-related knowledge* 4 items with dichotomous response options. From CDC’s Behavioral Risk Factor Surveillance System [145]	Self-reported previous HIV testing	*Perceived risk of HIV infection* *Ns*. *p* = 0.12 *HIV-related knowledge* *Ns*, *p* = 0.86
Ford et al. [74]	U.S.A. Routine testing at STD clinic. Adults ≥18 years. Self-reported Black ethnicity Seeking STI diagnosis or screening for possible STI	Cross-sectional Convenience sampling Response rate 87 % *n* = 373 163 (43.7 %) males, 210 (56.3 %) females	*Perceived racism* 10 items on Likert scale Adapted from [146, 147] *α* ≥ 0.70 *Stress coping mechanisms* 1 item, responses categorised as healthful (e.g., ‘exercise’), passive (e.g. ‘sleeping’), or negative (e.g. ‘drinking’)	HIV testing via blood draw, as recorded by the clinic	*Perceived racism* Higher perceived racism associated with higher likelihood of HIV testing (OR 1.68, 1.17–2.40; AOR 1.64, 1.07–2.52) *Stress coping mechanisms* Healthful coping not associated with testing (AOR 1.08, 0.91–1.27) Passive coping not associated with testing (AOR 0.89, 0.78–1.01) Negative coping not associated with testing (AOR 0.96. 0.89–1.05)
Ford et al. [42]	U.S.A. Urban area Older adults aged ≥50 years No previous diagnosis of HIV infection	Cross-sectional Stratified sampling by public health venue and time. *n* = 226. 146 (64.6 %) males, 80 (35.4 %) females. Age 50–85 years	*Belief in AIDS-related conspiracy theories* 4 items on Likert scale From [148], *α* = 0.84 **Mistrust in government**: 3 items on Likert scale. From [149], *α* = 0.63 *Perceived future risk of HIV* 8 items. Likert. Adapted [150], *α* = 0.59 *HIV-related knowledge* 8 true/false items for current study	HIV testing in last 12 months	*Belief in AIDS-related conspiracy theories* Belief associated with higher likelihood of testing in last 12 months (OR 1.86, 1.03–3.34; AOR 1.94, 1.05–3.60) *Mistrust in government* Mistrust associated ns with less likelihood of testing in last 12 months (OR 0.71, 0.45–1.11) but associated with testing in last 12 months in adjusted analysis (AOR 0.43, 0.26–0.73) *Perceived future risk of HIV* *Ns*, *p* = 0.33 *HIV-related knowledge* *Ns*, *p* = 0.07
Gu et al. [43]	China. Urban area Voluntary counselling and testing (VCT) MSM based in Hong Kong Aged ≥18 years	Cross-sectional Convenience sampling Response rate approximately 80 % for participants recruited from gay venues. *n* = 577	*HIV/STD-related knowledge* 3 items with dichotomous response options. Developed for current study **Theory of Planned Behaviour (TPB)** *Attitudes* 3 items on Likert scale: *Subjective norms* 3 items on Likert scale. *Perceived behavioural control* 3 items on Likert scale: *Behavioural intentions* 1 item on Likert scale All TPB measures developed for current study *Level of fear of contracting HIV* 1 item on 10-point numeric rating scale *Perceived discrimination towards local MSM* 1 item on Likert scale	Self-reported uptake of VCT: Last 12 months Lifetime	*HIV/STD-related knowledge* Positive association between >2 correct responses and VCT in last 12 months (OR 2.38, *p* < 0.001; AOR 2.35, 1.64-3.37, *p* < 0.05) and lifetime (OR 2.36, *p* < 0.001; AOR 2.45, 1.72–3.49, *p* < 0.001). *Attitudes.* ‘*It is necessary to take up antibody testing regularly*’ –associated with VCT in last 12 m (OR 1.87, *p* < 0.01; AOR 1.69, 1.14–2.52, *p* < 0.01) and lifetime (OR 1.70, *p* < 0.01; AOR 1.51, 1.03–2.21, *p* < 0.05). ‘*HIV antibody testing can protect you’—*associated with VCT in last 12 m (OR 2.35, *p* < 0.05; AOR 2.12, 1.23–3.68, *p* < 0.01) and lifetime (OR 2.24, *p* < 0.001; AOR 2.06, 1.26–3.37, *p* < 0.01). ‘*There are good testing services in Hong Kong’—*associated with VCT in last 12 m(OR 1.69, *p* < 0.01; AOR 1.69, 1.19–2.41, *p* < 0.01) and lifetime (OR 1.70, *p* < 0.01; AOR 1.56, 1.09–2.23, *p* < 0.05) *Subjective norms* ‘Perceived prevalence of MSM who have been tested for HIV’: perceiving a higher prevalence of testing (≥21 %) associated with VCT in last 12 m (OR 3.60, *p* < 0.001; AOR 3.69, 2.04–6.68, *p* < 0.001) and lifetime (OR 3.43, *p* < 0.001; AOR 3.68, 2.02–6.70, *p* < 0.001).‘Perceived that >50 % MSM peers would NOT test in the future’—negatively associated with VCT in last 12 m (OR 0.59, *p* < 0.01, AOR 0.56, 0.39–0.80, *p* < 0.01) and lifetime (OR 0.71, *p* < 0.05; AOR 0.68, 0.48–0.97, *p* < 0.05). ‘*Most MSM gave positive comments on HIV testing’—*associated with VCT in last 12 m (OR 1.63, *p* < 0.01; AOR 1.66, 1.16–2.36, *p* < 0.01) and lifetime (OR 1.92, *p* < 0.001; AOR 1.88, 1.31–2.71, *p* < 0.001). *Perceived behavioural control* ‘You can take up HIV testing if you wish’ –associated with VCT in last 12 m (OR 1.96, *p* < 0.05; AOR 1.66, 1.01–2.91, *p* < 0.05) and lifetime (OR 2.07, *p* < 0.01; AOR 1.74, 1.04–2.90, *p* < 0.05). ‘You have confidence you will take up HIV testing regularly’: associated with VCT in last 12 m(OR 4.60, *p* < 0.001; AOR 4.71, 3.22–6.89, *p* < 0.001) and lifetime (OR 3.51, *p* < 0.001; AOR 3.31, 2.25–4.87, *p* < 0.001).‘You will take up HIV testing even if afraid to know results’: associated with VCT in last 12 m (OR 4.19, *p* < 0.001; AOR 3.85, 2.44–6.08, *p* < 0.001) and lifetime (OR 4.37, *p* < 0.001; AOR 4.00, 2.66–6.00, *p* < 0.001). *Behavioural intentions* Any chance of testing in 6 m associated with VCT in last 12 m (OR 3.08, *p* < 0.001; AOR 2.88, 1.96–4.23, *p* < 0.001) and lifetime (OR 2.24, *p* < 0.001; AOR 2.12, 1.47–3.04, *p* < 0.001). *Level of fear of contracting HIV* Associated with decreased VCT in last 12 m (OR 0.63, *p* < 0.05; AOR 0.63, 0.40–0.99, *p* < 0.05) and lifetime (OR 0.64, *p* < 0.05) *Perceived discrimination* *Ns* with 12 m VCT (OR 0.90; AOR 0.78, CI 0.54–1.13), with lifetime VCT (OR 0.73, *p* < 0.1; AOR 0.65, 0.45–0.95, *p* < 0.05)

References	Location, inclusion/ exclusion and testing context	Design and sampling	Measurement of psychological variables	Measure of testing behaviour	Associations between psychological variables and testing
Hendriksen et al. [75]	48 communities in Tanzania, Zimbabwe, South Africa (Vulindlela, Soweto) and Thailand. Aged 18–32 living in selected households	Cross-sectional Stratified sampling by community *n* = 14,818 6638 (44.8 %) males, 8180 (55.2 %) females	*Perceived social norms* 6 items on Likert scale Developed for current study *Stigma* 19 items on Likert scale From [151]. 3 dimensions: negative attitudes towards PLWH (*α* = 0.82), perceived discrimination (*α* = 0.81), equity (*α* = 0.68)	Self-reported previous HIV testing	*Perceived social norms Ns* (for all sites): Tanzania (OR 0.77, 0.40–1.48); Zimbabwe (OR 1.82, 0.81–4.10); Vulindlela (OR 0.57, 0.26–1.22); Soweto (OR 0.82, 0.53–1.25); Thailand (OR 1.01, 0.54–1.91) *Stigma* In Thailand, high stigma significantly associated with lower levels of testing (OR 0.43, 0.29–0.64, *p* < 0.001). Tanzania (OR 0.71, 0.42–1.17, *ns*) Zimbabwe (OR 0.56, 0.25–1.25, *ns*); Vulindlela (OR 0.86, 0.46–1.59, *ns*) Soweto (OR 0.85, 0.57–1.27, *ns*)
Hong et al. [44]	Guangxi, China Urban area Female sex workers (FSW)	Cross-sectional Cluster sampling Response rate approximately 70 %. *n* = 1022 1022 (100 %) females. Age range 15–50 years 862 (84.4 %) Han Chinese, 160 (15.6 %) non-Han	*Self-rated HIV knowledge* 1 item on Likert scale *Perceived future risk of HIV* 1 item on Likert scale	Self-reported previous HIV testing	*Self-rated HIV knowledge* Higher self-rated knowledge associated with higher likelihood of testing (AOR 3.25, 1.95–5.55, *p* < 0.001) *Perceived future risk of HIV* *Ns* (AOR 0.70, 0.47–1.05)
Hoyt et al. [45]	U.S.A. Rural and urban areas. MSM, primary residence in selected areas in Arizona. Aged ≥18 years	Prospective cohort. Convenience and snowball sampling *n* = 394. Mean age 37 years (*SD* = 11.35). 299 (76 %) White, 51 (13 %) Latino, 20 (5 %) African American, 16(4 %) Native American, 8 (2 %) Asian American Attrition rate 38 %	**Institutional mistrust** *Systematic discrimination* 4 items on Likert scale*, α* = 0.86 *Organisational suspicion* 4 items on Likert scale*, α* = 0.77 *Conspiracy beliefs* 3 items on Likert scale*, α* = 0.76 Developed for current study *Perceived susceptibility* 3 items on Likert scale From [152–154], *α* = 0.84	Self-reported previous HIV testing	**Institutional mistrust** *Systematic discrimination* Higher perceived systematic discrimination associated with lower likelihood of testing (AOR 1.61, 1.14–2.28, *p* < 0.01) *Organisational suspicion* *Ns* (AOR 1.01, 0.67–1.52) *Conspiracy beliefs* *Ns* (AOR 0.78, 0.50–1.22) *Perceived susceptibility* *Ns* for ethnic minority MSM (*r* = −0.1) and White MSM (*r* = 0.04)

References	Location, inclusion/exclusion and testing context	Design and sampling	Measurement of psychological variables	Measure of testing behaviour	Associations between psychological variables and testing
Huang et al. [46]	China Urban area MSM aged ≥18 years	Cross-sectional Respondent-driven sampling *n* = 404 Mean age 29.6 years (*SD* = 10.4) 386 (96 %) Han, 16 (4 %) non-Han. 200 (49.5 %) money boys, 204 (50.5 %) general MSM	*Perceived risk of current HIV infection.* 1 item with dichotomous response options *Sexual Attitudes* [155]. Measures sexual permissiveness/responsibility, *α* = 0.75 (sex workers), *α* = 0.81 (general MSM) *Loss of Face* [156]. Measures perceptions of social propriety, self-discipline and social status. 21 items on Likert scale. *α* = 0.71 (sex workers), *α* = 0.78 (general MSM) *Knowledge of testing site* 1 item with dichotomous response options *HIV-related knowledge* 8 items with dichotomous response options Developed for current study	Self-reported previous HIV testing	*Perceived risk of current HIV infection* *Ns* (AOR 0.90, 0.60–1.60) *Sexual Attitudes* *Ns*. *p* = 0.26 *Loss of Face* *Ns*, *p* = 0.26 *Knowledge of testing site* Not knowing a testing site significantly associated with never testing (AOR 5.50, 2.70–11.30, *p* < 0.05) *HIV-related knowledge* Lower knowledge significantly associated with never testing (AOR 0.80, 0.70–0.90, *p* < 0.05)
Johnston et al. [47]	South Africa. Urban area. VCT. Black males ≥18 years old. >1 sexual partner in last 3 months. Partner either <24 years old or ≥ 3 years younger than participant	Cross-sectional Respondent-driven sampling *n* = 421 Age range 18–62 years	*Perceived risk of current HIV infection* 1 item on Likert scale	Acceptance of VCT at study site	*Perceived risk of current HIV infection* *Ns*: ‘*Somewhat likely infected’* (ref. ‘*very unlikely’*)—OR 1.40; AOR 1.40, *p* = 0.18); ‘*Very likely infected*’ (ref. ‘*very unlikely’*): OR 1.50; AOR 1.80, *p* = 0.09
Kakoko et al. [76]	Tanzania Urban and rural areas Primary school teachers in selected districts (districts selected on availability of testing services)	Cross-sectional Convenience sampling Response rate 94 % *n* = 918 315 (34.29 %) males, 603 (65.7 %) females Age range 21–59 years	*Self-rated health status* 1 item on Likert scale *Intention to test for HIV* 3 items on Likert scale Developed for current study *α* = 0.75 *Perceived susceptibility to HIV* 4 items. Likert.Developed for current study.*α* = 0.75 *Affordability of HIV testing* 1 item on Likert scale *Perceived accessibility of HIV testing,* 1 item on Likert scale *HIV-related stigma* 1 item on Likert scale *Absence of cure for HIV/AIDS* 1 item on Likert scale *Belief only people who suspect HIV infection should test* 1 item on Likert scale *Uncertainty about confidentiality* 1 item on Likert scale *Fear of dying earlier if diagnosed with HIV*: 1 item on Likert scale. Developed for current study	Self-reported previous HIV testing	*Self-rated health status*. Compared with ‘*poor/very poor*’ status, positively rated status associated with greater likelihood of testing: ‘*Fair*’—OR 2.36, 1.10–5.06, *p* < 0.05; AOR 2.22, 1.02–4.84, *p* < 0.05. ‘*Good/very good*’—OR 2.85, 1.32–6.17, *p* < 0.01; AOR 2.54, 1.15–5.62, *p* < 0.05 *Intention of testing for HIV Ns* (OR 1.25, 0.80–1.97; AOR 1.18, 0.75–1.88) *Perceived susceptibility to HIV Ns* (OR 0.99, 0.72–1.38; AOR 0.98, 0.78–0.88). *Affordability of HIV testing Ns* (OR 0.81, 0.58–1.12; AOR 0.80, 0.57–1.12) *Accessibility of HIV testing* Poor accessibility of testing sites associated with less likelihood of testing (OR 0.45, 0.28–0.78, *p* < 0.01; AOR 0.62, 0.40–0.98, *p* < 0.05) *HIV-related stigma* Low perceived stigma associated with greater likelihood of testing in univariate (OR 1.72, 1.23–2.40, *p* < 0.05) but not multivariate analysis (AOR 0.92, 0.60–1.42, *ns*) *Absence of cure for HIV/AIDS* Disagreement with belief in no cure for HIV/AIDS associated with higher likelihood of testing (OR 2.19, 1.56–3.06, *p* < 0.01; AOR 1.00, 1.01–2.33, *p* < 0.05) *Belief only people who suspect HIV infection should test* Belief associated with less likelihood of testing (OR 0.63, 0.46–0.88, *p* < 0.01; AOR 0.52, 0.33–0.81, *p* < 0.01) *Uncertainty about confidentiality* Belief that test results are confidential associated with greater likelihood of testing in univariate (OR 1.51, 1.08–2.11, *p* < 0.05) but not multivariate analysis (AOR 0.85, 0.57–1.26, *ns*) *Fear of dying earlier if diagnosed with HIV* Less fear associated with >likelihood of testing (OR 2.87, 2.04–4.03, *p* < 0.01; AOR 1.93, 1.26–2.95, *p* < 0.05)
Kalichman and Simbayi [77]	South Africa Urban area Individuals living in selected township	Cross-sectional Convenience sampling *n* = 500 224 (44.8 %) males, 276 (55.2 %) females Median age range 21–25 years 490 (98 %) Black ethnicity	*HIV-related knowledge* 12 items with dichotomous response options. Adapted from [140], *α* = 0.70 *HIV testing attitudes* 5 items with dichotomous response options. Adapted from [157] *HIV-related stigma* 13 items with dichotomous response options. Adapted from [158]	Self-reported previous HIV testing	*HIV-related knowledge* *Ns* (AOR 0.49, 0.15–1.58) *HIV testing attitudes* *‘Getting tested for HIV helps people feel better’—*agreement associated with testing (AOR 2.9, *p* < 0.01) ‘*Getting tested for HIV helps people from getting HIV’—*agreement associated with testing (AOR 2.2, *p* < 0.01) ‘*People in my life would leave me if I had HIV’—*agreement negatively associated with testing (AOR 0.5, *p* < 0.01) ‘*People who test positive should hide it from others’—*agreement negatively associated with testing (AOR 0.4, *p* < 0.01) ‘*I would rather not know I had HIV’—*agreement negatively associated with testing (AOR 0.5, *p* < 0.01) *HIV-related stigma* Individuals with stigmatising beliefs less likely to have tested: ‘*People who have AIDS are dirty*’*—*AOR 0.30, *p* < 0.01 ‘*People who have AIDS should be ashamed*’*—*AOR 0.40, *p* < 0.01

References	Location, inclusion/exclusion and testing context	Design and sampling	Measurement of psychological variables	Measure of testing behaviour	Associations between psychological variables and testing
Kaufman et al. [115]	11 districts, Malawi. Adults aged ≥18 years Sexually experienced	Intervention (individual and community behaviour change). Stratified sampling by district and exposure group *n* = 594. 271 (45.6 %) males, 323 (54.4 %) females. Mean age 29.1 years (males), 27.7 years (females)	*HIV-related knowledge* 11 items with dichotomous response options *α* = 0.63 *Self-efficacy* 9 items on Likert scale *α* = 0.73 *Perceived risk of HIV (to self and family)* 3 items on Likert scale *α* = 0.81	Self-reported HIV testing in last year	*HIV-related knowledge* AOR 1.05, 0.96–1.16, *ns* *Self-efficacy* AOR 0.99, 0.94–1.05, *ns* *Perceived risk of HIV* AOR 0.98, 0.93–1.02, *ns* IVs adjusted for baseline scores pre-intervention exposure Intervention exposure associated with increases in HIV-related knowledge (β = 0.20, 0.06–0.34, *p* < 0.01) and self-efficacy (β = 0.35, 0.08–0.62, *p* < 0.01) Intervention exposure associated with testing (AOR 1.40, 1.16–1.70, *p* < 0.001)
Kellerman et al. [78]	U.S.A. Urban. Individuals at high risk for HIV (MSM, IDU, heterosexual individuals recruited from gay bars, outreach, STD clinics). Aged ≥18 years Resident in selected state Self-reported HIV-negative	Cross-sectional Convenience sampling *n* = 1711 1270 (74.2 %) males, 441 (25.8 %) females 18–44 years 757 (44.2 %) White, 385 (22.5 %) African American, 389 (22.7 %) Hispanic	*HIV testing knowledge* 4 items on Likert scale: Developed for current study *HIV testing fear* 4 items on Likert scale: Developed for current study	Self-reported previous HIV testing	*HIV testing knowledge ‘If I had HIV I would tell my sex partners’—*agreement positively associated with testing, *p* < 0.0001. *‘People I have sex with want to know my HIV status’—*agreement positively associated with testing, *p* < 0.0001) *‘Medical care can help sick people with HIV to be healthier’—*among *MSM*, agreement positively associated with testing, *p* < 0.0001.*‘Medical care can help well people with HIV to be healthier’—*among *MSM*, agreement positively associated with testing, *p* < 0.0001 *HIV testing fear* ‘*I could handle finding out I had HIV’—*among *MSM*, agreement positively associated with testing, *p* < 0.0001.‘*I* *would rather not know I had HIV until I had to*’– agreement negatively associated with testing, *p* < 0.0001. *‘If I had HIV, I wouldn’t tell people’*: agreement negatively associated with testing, < 0.001). *‘If I had HIV, my sex life would be ruined’—*agreement negatively associated with testing, *p* < 0.001
Knox et al. [49]	South Africa Urban area MSM living in greater Pretoria 18–40 years	Cross-sectional Convenience sampling *n* = 300 Age range 18–40 years 199 (66.3 %) Black, 101 (33.7 %) White ethnicity	*HIV-related knowledge* 15 items with dichotomous response options. Adapted from [159, 160] **Sexual minority stress** *Internalised homophobia* *Sexual orientation*-*based discrimination (lifetime and in past year)* Adapted from [161, 162]	Self-reported previous HIV testing. Ever tested. Tested in past year	*HIV-related knowledge* Low HIV-related knowledge negatively associated with ever testing, AOR 0.90, 0.80–1.00, *p* = 0.05. No association with testing in past year vs. testing >1 year ago, *p* = 0.99 *Sexual minority stress Internalised homophobia.* Negatively associated with ever testing, *p* = 0.02. Negatively associated with testing in past year vs. testing >1 year ago, AOR 0.63, 0.43–0.94, *p* = 0.02. *Sexual orientation*-*based discrimination (lifetime and in past year)*. No association between lifetime discrimination and ever testing, *p* = 0.34, or testing in past year vs >1 year ago, *p* = 0.11. No association between discrimination in past year and ever testing, *p* = 0.95) Discrimination in past year associated with testing in past year vs. testing >1 year ago, *p* = 0.02

References	Location, inclusion/exclusion and testing context	Design and sampling	Measurement of psychological variables	Measure of testing behaviour	Associations between psychological variables and testing
Koku [50]	Ghana. Urban and rural areas Women 15–49 years. Sexually active in last 12 months	Cross-sectional Stratified sampling by enumeration area *n* = 3766	*HIV-related knowledge* 5 items with dichotomous response options. *Personal stigma* 4 items with dichotomous response options,	Self-reported previous HIV testing	*HIV-related knowledge* High level of knowledge associated with higher likelihood of testing (AOR 1.64, 0.28–0.77, *p* < 0.01) *Personal stigma‘I would keep a relative’s HIV infection a secret’—ns* (AOR 1.02, 0.69–1.51).‘*A female teacher with AIDS should not teach’—*agreement associated with less likelihood of testing (AOR 0.74, 0.40–0.88, *p* < 0.01)
Lau and Wong [79]	China, Urban area. Male. Reported sexual intercourse with female sex worker (FSW) in past 6 months	Cross-sectional *n* = 250 Age range 18–45 + years	*Perceived future risk of HIV* 1 item. Dichotomous response. *Perceived efficacy of condom use* 1 item on Likert scale *Knowledge about modes of HIV transmission* 1 open-ended question, number of correct answers coded.	Self-reported HIV testing in past 6 months	*Perceived risk of contracting HIV* *Ns* (OR 1.47, 0.74–2.94, *p* = 0.27) *Perceived efficacy of condom use* *Ns* (OR 1.42, 0.31–6.47, *p* = 0.99) *Knowledge about modes of HIV transmission* *Ns* (OR 1.63, 0.68–3.91, *p* = 0.38)
Lofquist [51]	Kenya. Urban areas One of at-risk populations: FSW, low-income women (LIW), men on worksites (MOW), and policemen Aged 15–49 years	Cross-sectional Cluster sampling Response rate 99 % for all populations FSW: *n* = 1749 LIW: *n* = 2076 MOW: *n* = 2097 Policemen: *n* = 568	**Health Belief Model** *Perceived susceptibility* *Perceived risk for contracting HIV* 1 item on Likert scale *Knowledge of HIV prevention* 3 items. Dichotomous response. Developed for current study *Perceived severity.* 1 item with dichotomous response options *Perceived barriers. HIV/AIDS*-*related myths.* 6 items with dichotomous response options *Perceived stigma*. 6 items with dichotomous response options *Confidentiality availability.* 1 item with dichotomous response options Developed for current study *Perceived benefits. Utility of VCT if HIV*-*negative*. 7 items with dichotomous response options *Utility of VCT if HIV-positive. 9 items with dichotomous response options. Developed for current study* *Knows someone with HIV.* 1 item with dichotomous response options.	Self-reported previous HIV testing	**Health Belief Model** ***Perceived susceptibility.*** *Perceived risk for contracting HIV*. FSW: Moderate/high perceived risk negatively associated with testing (AOR 0.68, *p* < 0.05); LIW: *Ns* (AOR 0.53); MOW: *Ns*(AOR 0.96). Policemen: *Ns* (AOR 0.86) *Knowledge of HIV prevention*: FSW: *Ns* (AOR 0.85); LIW: *Ns* (AOR 1.27) MOW: *Ns* (AOR 0.89); Policemen: *Ns* (AOR 0.80) *Perceived severity.* FSW: *Ns* (AOR 0.71); LIW: *Ns* (AOR 0.83); MOW: *Ns* (AOR 0.73). Policemen: *Ns* (AOR 0.58) *Perceived barriers. HIV/AIDS*-*related myths*. FSW: higher level of myths negatively associated with testing (AOR 0.72, *p* < 0.05); LIW: *Ns* (AOR 1.38); MOW: *Ns* (AOR 1.32); Policemen: *Ns* (AOR 0.99) *Perceived stigma.* FSW: *Ns* (AOR 1.10); LIW: *Ns* (AOR 0.87); MOW: *Ns* (AOR 0.82); Policemen: *Ns* (AOR 1.01) *Confidentiality availability*. FSW: *Ns* (AOR 0.72); LIW: belief confidential testing is unavailable associated with less likelihood of testing (AOR 0.39, *p* < 0.001); MOW: belief confidential testing is unavailable associated with less likelihood of testing (AOR 0.41, *p* < 0.01); Policemen: *Ns* (AOR 0.72) *Perceived benefits. Utility of VCT if HIV*-*negative*. FSW: *Ns* (AOR 0.95); LIW: significant negative association with testing (AOR 0.74, *p* < 0.05); MOW: perceiving a higher level of utility of VCT if HIV-negative was negatively associated with testing (AOR 0.75, *p* < 0.05); Policemen: *Ns* (AOR 0.99). *Utility of VCT if HIV*-*positive.* FSW: *Ns* (AOR 0.96); LIW: *Ns* (AOR 0.95); MOW: *Ns* (AOR 1.20); Policemen: *Ns* (AOR 1.04) *Knows someone with HIV* FSW: *Ns* (AOR 1.01); LIW: *Ns* (AOR 1.10); MOW: *Ns* (AOR 1.43); Policemen: *Ns* (AOR 1.70)

References	Location, inclusion/exclusion and testing context	Design and sampling	Measurement of psychological variables	Measure of testing behaviour	Associations between psychological variables and testing
Ma et al. [52]	China Urban area Heterosexual attendees of four STD clinics Sexually active Aged >14 years	Cross-sectional Convenience sampling Response rate 78.8 % *n* = 823 517 (62.8 %) males, 306 (37.2 %) females 342 (41.6 %) aged < 30 years	*Perceived risk of HIV* *Perceived risk of STD* *HIV-related knowledge* 4 items with dichotomous response options. *α* = 0.83 *Awareness that county has established VCT site*	Self-reported HIV testing in last 6 months	*Perceived risk of HIV* For men, significant association with HIV testing (OR 4.04, 1.60–10.16, *p* = 0.003). For women, ns (OR 0.77, 0.09–6.53, *p* = 0.81) *Perceived risk of STD* For men, ns (OR 0.59, 0.32–1.08, *p* = 0.09). For women, ns (OR 0.94, 0.50–1.76, *p* = 0.84). *HIV-related knowledge* For men, getting 1–3/4 correct, and 4/4 correct (reference: 0/4 correct) significantly associated with testing (OR 5.93, 1.35–26.04, *p* = 0.02; OR 9.90, 2.31–42.33, *p* = 0.002, respectively). For women, ns association between getting 1–3/4 correct and testing (OR 1.13, 0.51–2.50, *p* = 0.77; but significant association between 4/4 correct items and testing (OR 3.16, 1.42–7.03, *p* = 0.005). *Awareness that county has established VCT site* For men, awareness associated with testing (OR 2.99, 1.61–5.56, *p* = 0.001) For women, awareness associated with testing (OR 2.75, 1.50–5.06, *p* = 0.001)
Mack and Bland [91]	U.S.A. Rural and urban areas Voluntary testing. Aged ≥ 50 years	Cross-sectional Simple random sampling *n* = 21132. Age range 50–64 years	*Perceived future risk of HIV* 1 item on Likert scale. 1996 Behavioral Risk Factor Surveillance System (BRFSS)	Self-reported voluntary HIV testing	*Perceived future risk of HIV* Perceived medium/high risk associated with higher likelihood of voluntarily testing (AOR 0.60, *p* = 0.002). Perceived low risk *ns* (AOR 0.86, *p* = 0.08)
MacPhail et al. [90]	South Africa Rural and urban areas Adolescents aged 15–24 years Sexually experienced	Cross-sectional Stratified sampling by enumeration area *n* = 7655. 3609 (47 %) males, 4058 (53 %) females 6583 (86 %) Black ethnicity	*Knowing someone with HIV/AIDS* *Knowing someone who died of HIV/AIDS* *Rejecting a friend with HIV*	Self-reported previous HIV testing	*Knowing someone with HIV/AIDS* Among men, *ns* (AOR = 1.06, 0.73–1.56, *p* = 0.75) Among women, *ns* (AOR 1.20, 0.95–1.50, *p* = 0.12) *Knowing someone who died of HIV/AIDS* Among men, significant association with testing (AOR 1.68, 1.14–2.47, *p* = 0.01). *Rejecting a friend with HIV* Among men, *ns* (AOR 0.63, 0.34–1.18, *p* = 0.15) Among women, *ns* (AOR 0.63, 0.39–1.03, *p* = 0.067)
Maguen et al. [80]	U.S.A. Urban area Lesbian, gay or bisexually oriented students	Cross-sectional Convenience sampling. *n* = 117. 63 (52 %) males, 53 (44 %) females, 1 (4 %) trans. Mean age: males 20.1 years, females 19.9 years. 86 (73.5 %) White, 13 (11.1 %) Black, 6 (5.1 %) Latino, 5 (4.3 %) Asian, 5 (4.3 %) Biracial, 1 (0.9 %) other.	*HBM variables* *Perceived susceptibility* 1 item on Likert scale: ‘*I am so sure I don’t have the AIDS virus that I don’t have to be tested.*’ *Perceived barriers to HIV testing* 11 items on Likert scale Adapted from [163] *α* = 0.85	Self-reported previous HIV testing	*Perceived susceptibility* Lower perceived susceptibility associated with less likelihood of testing (AOR 3.45, *p* < 0.01) *Perceived barriers to HIV testing* Higher perceived barriers associated with less likelihood of testing (AOR 1.15, *p* < 0.05) *HBM variables together* accounted for an additional 18 % variance of model (over and above demographic/behavioural factors), *R* ^2^ = 0.18, *χ* ^2^ = 24.29, *p* < 0.01

References	Location, inclusion/exclusion and testing context	Design and sampling	Measurement of psychological variables	Measure of testing behaviour	Associations between psychological variables and testing
Massari et al. [53]	France Urban area Aged ≥18 years Living in selected households in each census block	Cross-sectional Systematic random sampling Response rate 71 %. *n* = 3023 1423 (47.1 %) males, 1600 (52.9 %) females Age range 18–60 years. 2068 (68.4 %) French, 536 (17.7 %) French/other ethnicity parents, 419 (13.9 %) other ethnicity	*Perceived risk of HIV* 1 item with dichotomous response options *Perceived social support* 1 item with dichotomous response options	Self-reported previous HIV testing	*Perceived risk of HIV* In men, low perceived risk for HIV associated with never testing (AOR 1.71, 1.23–2.38, *p* = 0.05). *Ns* in women (tested vs. never tested, *p* = 0.29) *Perceived social support* *Ns* in tested and untested men (tested vs. never tested, *p* = 0.96), and women (tested vs. never tested, *p* = 0.12)
Matovu et al. [54]	Uganda. Urban and rural areas. Individuals in long-term relationships (duration at least 1 year) Women aged 18–49, men aged 18–54	Case–control Stratified sampling by catchment area *n* = 787. 359 (45.6 %) males, 428 (54.4 %) females. 296 (37.6 %) aged 18–24 years	*Belief HIV discordance is possible* 1 item with dichotomous response options *Perceived risk of HIV* 1 item on Likert scale	Self-reported previous HIV testing (individual) Self-reported uptake of couples’ HCT	*Belief HIV discordance is possible* Belief significantly associated with previous (individual) testing (OR 1.94, 1.37–2.75; AOR 1.77, 1.20–2.63, *p* < 0.05) **Perceived risk of HIV** *Ref: Very likely to be at risk*. *Ns* association between unknown risk and previous couples’ HCT (OR 1.63, 0.92–2.87; AOR 0.64, 0.32–1.29) *Ns* association between very unlikely risk and previous couples’ HCT in adjusted analysis (OR 2.25, 1.32–3.83; AOR 1.64, 0.86–13.13) *Ns* association between a limited risk and previous couples’ HCT (OR 1.27, 0.85–1.91; AOR 1.38, 0.83–2.28)
McGarrity and Huebner [55]	U.S.A. Urban area HIV-negative MSM	Prospective cohort (over 6 months). Convenience and snowball sampling *n* = 487. 18–72 years (mean age 35.7 years). 362 (74.4 %) White, 67 (13.8 %) Latino, 56 (11.5 %) other ethnicity. Attrition rate 31 %	*Intention to test for HIV in next 6* *months* 1 item on Likert scale	Self-reported HIV testing during 6 month follow-up period	*Intention to test for HIV in next 6* *months* Significant association between intention and testing (AOR 1.32, 1.13–1.54, *p* < 0.001) Socioeconomic status (SES) moderated association between intention and behaviour, with intention being a significant predictor of testing behaviour in high SES individuals (AOR 1.53, *p* < 0.001), but not low SES individuals (AOR 1.14, *ns*)
McNaghten et al. [81]	Zimbabwe Rural and urban areas Provider-initiated testing Individuals aged 15–29 years Living in selected households in census areas	Cross-sectional Stratified random sampling by location. 76 % response rate among females, 72 % among males *n* = 9010. 4200 (46.6 %) males, 4810 (53.4 %) females	*Perceived risk of HIV*	Provision of blood specimen for HIV test at time of study	*Perceived risk of HIV* *Ns* in women (‘*no risk’*: *p* = 0.06). *Ns* in men (‘*no risk’*: *p* = 0.18).
Melo et al. [93]	Brazil Individuals receiving care at mental health institutions or outpatient clinics Aged ≥18 years	Cross-sectional Simple random sampling Response rate 89.6 %, *n* = 2475 1147 (48.2 %) males, 1233(51.8 %) females	*HIV-related knowledge* 10 items with dichotomous response options From [93] *Perceived risk of HIV* 1 item on Likert scale	Self-reported previous HIV testing	*HIV-related knowledge* Higher HIV-related knowledge associated with increased likelihood of testing (OR 2.93, 2.11–4.06, *p* < 0.001; AOR 1.65, 1.24–2.18, *p* < 0.001). *Perceived risk of HIV* ‘*Not known’* (ref. ‘*high risk’*)*—*associated with less likelihood of testing (OR 0.48, 0.34–0.67, *p* < 0.001; AOR 0.57, 0.43–0.77, *p* < 0.001). ‘*No risk’—*associated with less likelihood of testing in crude but not adjusted analysis (OR 0.62, 0.43–0.88, *p* = 0.009; AOR 0.75, 0.54–1.04, *ns*). ‘*Medium risk’—ns* (OR 0.83, 0.59–1.17; AOR 0.83, 0.59–1.16)
Menser [97]	U.S.A. Urban area Students	Cross-sectional Convenience sampling. *n* = 440 174 (40 %) males, 261 (60 %) females Age range 18–55 years (*M* = 19.5 years). 355 (83.1 %) Caucasian, 31 (7.3 %) African American, 27 (6.3 %), Asian/Pacific Islander, 6 (1.4 %) Hispanic, 8 (1.9 %) other	**Pro-HIV testing items** *Security and responsibility* 3 items on Likert scale: e.g. *‘Taking an HIV test would give you a sense of security’.* Adapted from [164] *Con-HIV testing items* *Fear of needles* 1 item on Likert scale Adapted from [164] *Perceived risk of HIV* 4 items on Likert scale From [164]	Self-reported previous HIV testing	**Pro-HIV testing items** *Security and responsibility* Significantly associated with testing, *p* = 0.006 **Con-HIV testing items** *Fear of needles* Significantly associated with no testing, *p* = 0.02 *Perceived risk of HIV* Significantly associated with testing, *p* < 0.05
Mirkuzie et al. [58]	Ethiopia. Urban area. Antenatal HIV testing Women not known to be HIV-positive Attending antenatal care for first time in pregnancy	Prospective cohort Convenience sampling. 96.5 % response rate. *n* = 3033. Age range 15–25+ years. Attrition rate 3.5 %	*Prevention of mother-to-child transmission (PMTCT) knowledge* 5 items with dichotomous response options. Developed for current study **TPB constructs** *Intention to test for HIV* 3 items on Likert scale *Perceived barriers* 4 items on Likert scale Developed for current study	Testing for HIV in follow-up period (clinical records)	*Prevention of mother-to-child transmission (PMTCT) knowledge* PMTCT knowledge ns associated with testing (AOR 0.66, 0.38–1.16) **TPB constructs** *Intention to test for HIV* Stronger intention associated with increased likelihood of testing (AOR 2.38, 1.45–3.85) *Perceived barriers* Lower perceived barriers ns associated with testing (AOR 1.41, 0.83–2.38)
Norman and Gebre [89]	Jamaica Urban area University students Sexually experienced	Cross-sectional Convenience sampling. *n* = 961 309 (32.2 %) males, 652 (67.8 %) females Mean age 28.2 years (SD = 9.1)	*Perceived future risk of HIV* 1 item on Likert scale *Personal awareness of HIV* Participants asked if knew someone infected with HIV or had died from AIDS. 1 item with dichotomous response options	Self-reported previous HIV testing	*Perceived future risk of HIV* Ns association with testing (*p* = 0.88; AOR 1.25, 0.92–1.70, *p* = 0.16). *Personal awareness of HIV.* Significant association with testing (*p* < 0.001; AOR 1.39, 1.02–1.90, *p* = 0.04)
Norman [82]	Jamaica Rural and urban areas Individuals living in selected households Aged 15–49 years	Cross-sectional Stratified random sampling by parish. *n* = 1800 914 (50.8 %) males, 886 (49.2 %) females Mean age 30.1 years (SD = 10.8)	*Perceived future risk of HIV* 1 item on Likert scale *Personal awareness of HIV* Participants asked if knew someone infected with HIV or had died from AIDS 1 item with dichotomous response options	Self-reported previous HIV testing	*Perceived future risk of HIV* Significant positive association with testing (OR 1.43, 1.15–1.77, *p* < 0.01; AOR 1.36, 1.09–1.70, *p* < 0.01) *Personal awareness of HIV* Significant positive association with testing (OR 1.54, 1.26–1.90, *p* < 0.001; AOR 1.39, 1.11–1.74, *p* < 0.01)
Norman et al. [83]	Puerto Rico Urban area Female Resident of Public Housing Department	Cross-sectional Convenience sampling *n* = 1138 Mean age 36.8 years (*SD* = 12.3)	*Perceived future risk of HIV* 1 item on Likert scale *Personal awareness of HIV* Participants asked if knew someone infected with HIV or had died from AIDS. 1 item with dichotomous response options *HIV-related knowledge* 21 items with dichotomous response options Developed for current study	Self-reported previous HIV testing	*Perceived future risk of HIV* Significantly associated with testing (AOR 1.60, 1.11–2.32, *p* < 0.05) *Personal awareness of HIV* Knowing family/friends with HIV/AIDS associated with testing (AOR 1.86, 1.19–2.92, *p* < 0.01). *HIV-related knowledge Ns* (AOR 1.02, 0.95–1.10, *ns*)
Pettifor et al. [59]	South Africa Urban area Attendees of STI, family planning and VCT clinic Aged ≥15 years	Cross-sectional Convenience sampling *n* = 198 Mean age 24.5 years 198 (100 %) Black African	*HIV-related stigma* *Blame/shame. 10 items on Likert scale* **Discrimination. 8 items on Likert scale** *Equity.* 5 items on Likert scale From [165] *α* = 0.71–0.86 [165] *Perceived norms* 7 items on Likert scale *Perceived availability of ARVs* 5 items on Likert scale	Self-reported previous HIV testing	**HIV-related stigma** *Blame/shame:* more shame associated with less likelihood of *testing (OR* *0.35, 0.16*–*0.78; AOR* *0.35, 0.16*–*0.77).* *Discrimination*: lower discrimination ns associated with testing (OR 1.18, 0.60–2.32) **Equity: high equity associated with testing (OR 2.85, 1.17–6.90; AOR 2.87, 1.20–6.86)** **Perceived norms.** ‘*Most people want to get tested for HIV’*: Disagreement associated with testing (OR 2.56, 1.23–5.37; AOR 2.59, 1.29–5.24). *‘Most people get tested only if they are sick’:* Agreement associated with testing (OR 4.91, 1.68–14.30, AOR 4.66, 1.70–12.76) **Perceived availability of ARVs** ‘*ARVs are easily available in the community’:* Ns associated with testing (OR 0.48, 0.20–1.13). *‘ARVs are affordable’:* Ns associated with testing (OR 1.72, 0.73–4.04)
Prati et al. [60]	Italy Rural and urban areas MSM aged >18 years who have had sex with a man in the previous 12 months	Cross-sectional Convenience sampling *n* = 14,409 Age range 18–79 years	*Internalised homophobia* From [166] *Awareness of HIV testing services* 1 item on Likert scale *HIV test self-efficacy* 1 item on Likert scale	Self-reported previous HIV testing: Never tested/tested in past year/tested >12 months ago	*Internalised homophobia* Higher homophobia ns associated with increased likelihood of never testing compared with testing in past year (AOR 1.00, 0.96–1.04); or increased likelihood of testing more than a year ago compared with testing in past year (AOR 1.04, 1.00–1.08) *Awareness of HIV testing services* Not knowing whether free HIV testing was available associated with increased likelihood of never testing compared with testing in past year (AOR 0.18, 0.15–0.21); and increased likelihood of testing more than a year ago compared with testing in past year (AOR 0.52, 0.44–0.61) *HIV test self-efficacy* Those who were ‘not at all confident’ were more likely to have never tested than tested in past year (AOR 5.01, 3.56–7.46); and had increased likelihood of testing more than a year ago than testing in past year (AOR 2.12, 1.16–3.87)
Ratcliffet al. [32]	U.S.A. Rural area Rapid HIV testing Female Using shelter services for intimate partner violence	Cross-sectional Convenience sampling. *n* = 112 Age range 1865 years. 21 (19 %) Caucasian, 85 (76 %) African American, 1 (0.8 %) Hispanic, 5 (4.5 %) other ethnicity	**HBM constructs** *Perceived susceptibility to HIV* 4 items on Likert scale From [167]. *α* = 0.84 [168] *Perceived severity.* 4 items on Likert scale. From [169] *Perceived benefits.* 4 items on Likert scale. From [170] *α* = 0.75 [171] *Perceived barriers.* 4 items on Likert scale. Adapted [170] *Self-efficacy* 10 items on Likert scale From [172], α = 0.76–0.90	Acceptance of rapid HIV test at time of study	**HBM constructs** *Perceived susceptibility to HIV* Significant association with testing (AOR 1.13, 1.13–1.27, *p* = 0.05) *Perceived severity* *Ns* (AOR 1.03, 0.86–1.06, *p* = 0.63) *Perceived benefits* *Ns* (AOR 0.95, 0.83–1.17, *p* = 0.56) *Perceived barriers* *Ns* (AOR 1.07, 0.93–1.20, *p* = 0.36) *Self*-*efficacy* *Ns* (AOR 1.00, 0.95–1.08, *p* = 0.82)

References	Location and testing context	Design and sampling	Measurement of psychological variables	Measure of testing behaviour	Associations between psychological variables and testing
Sabato et al. [61]	U.S.A. Students on health courses at selected universities	Cross-sectional *n* = 1874. 552 (29.5 %) males, 1322 (70.5 %) females. 16–54 years 1539 (82.1 %) Caucasian, 109 (5.8 %) African American, 120 (6.4 %). Asian- Pacific Islander, 106 (5.7 %) other	*HIV-related knowledge* 18 items with dichotomous response options. From [140]. *α* = 0.78 *Depression* 8 items on Likert scale From [142]. *α* = 0.86 [173] *Attribution style* 13 items numeric rating scale. From [174] *α* = 0.83 *Locus of control for sexual activities* Extent that participants see their sexual activities regulated by internal vs. external control. 11 items. Likert. From [175]. *α* = 0.76	Self-reported previous HIV testing	*HIV-related knowledge* *Ns* in men (AOR 1.05, 0.94–1.17, *p* = 0.35). Significant positive association with testing in women (*t* = −3.64, *p* < 0.01; AOR 1.15, 1.12–1.20, *p* = 0.03) *Depression* *Ns* in men (AOR 0.99, 0.92–1.07, *p* = 0.94), and women (*p* < 0.05; AOR 1.01, 0.96–1.04, *p* = 0.80). *Attribution style* *Ns* in men (AOR 1.01, 0.96–1.05, *p* = 0.70) and women AOR 0.97, 0.95–1.00, *p* = 0.08) *Locus of control for sexual activities* Greater internal control associated with greater likelihood of testing in men (*p* < 0.05; AOR 0.89, 0.82–0.97, *p* = 0.01) and women (*p* < 0.01; AOR 0.96, 0.91–1.00, *p* = 0.05)
Sambisa et al. [62]	Zimbabwe Rural and urban areas. Self/provider-initiated testing. Resident in selected households. Women aged 15–49 years Men aged 15–54 years Sexually active	Cross-sectional Stratified random sampling by cluster. Household response rate 95 %, individual response rate 90 % for women, 82 % for men *n* = 12154 5315 (43.7 %) males, 6839 (56.3 %) females	**Stigma towards PLWHA** *Social rejection* 3 items on Likert scale *Prejudiced attitudes* 2 items on Likert scale *Disclosure concerns* 2 items on Likert scale Developed for current study **Observed enacted stigma** Whether participant knows PLWHA and has observed discrimination against them 4 items on Likert scale Developed for current study **HIV-related knowledge** *Abstinence* *Being faithful* *Condom use* *Healthy*-*looking person can have HIV* 4 items with dichotomous response options Developed for current study *Perceived future risk of HIV* 1 item on Likert scale	Self-reported previous HIV testing: Self-initiated (SIT) Provider-initiated (PIT)	**Stigma towards PLWHA.** *Social rejection*. Female: associated with SIT [*ref.* *never testing*] (RRR 0.75, 0.63–0.89, *p* < 0.001), PIT (RRR 0.72, 0.62–0.85, *p* < 0.001). Male: Ns SIT (RRR 0.91, 0.75–1.11), PIT (RRR 0.78, 0.60–1.02, p > 0.05). *Prejudiced attitudes* F: Ns SIT (RRR 1.00, 0.83–1.20), PIT (RRR 0.98, 0.83–1.14). M: Ns SIT (RRR 0.93, 0.75–0.15), PIT (RRR 1.27, 0.98–1.67, p < 0.10). D*isclosure concerns.* F: *Ns* SIT (RRR 0.99, 0.80–1.19) PIT (RRR 1.07, 0.90–1.28). M: *Ns* SIT (RRR 0.89, 0.71–1.10) PIT (RRR 1.24, 0.89–1.73). *Observed enacted stigma.* Knowing PLWHA but not observing discrimination against them (ref. knows no PLWHA). F: association with SIT (RRR 1.32, 1.06–1.63, *p* < 0.01). *Ns* for PIT (RRR 1.11, 0.91–1.35). M: association with SIT (RRR 1.40, 1.12–1.74, *p* < 0.001). *Ns* for PIT (RRR 1.15, 0.85–1.57). *Knowing PLWHA and observing discrimination (ref. knows no PLWHA)* F: association with SIT (RRR = 1.43, 1.17–1.75, *p* < 0.001) and PIT (RRR 1.24, 1.04–1.49, *p* < 0.05). M: association with SIT (RRR 1.41, 1.22–1.77, *p* < 0.01) and PIT (RRR 1.57, 1.17–2.10, *p* < 0.01). HIV-related knowledge. *Abstinence.* F: knowledge abstinence prevents transmission *ns* for SIT (RRR 0.94, 0.74–1.81). Association with PIT (RRR 1.28, 1.04–1.58, *p* < 0.05). M: *Ns* for SIT (RRR 0.94, 0.69–1.27) and PIT (RRR 1.08, 0.71–1.64). *Being faithful*. F: Knowledge faithfulness prevents transmission *ns* for SIT (RRR 0.92, 0.74–1.16) and PIT (RRR 1.08, 0.88–1.32). M: association with SIT (RRR 1.45, 1.08–1.96, p < 0.05). *Ns* PIT (RRR 0.80, 0.56–1.13). *Condoms* F: knowledge condoms prevent transmission *ns* SIT (RRR 1.10, 0.89–0.37). Association with PIT (RRR = 1.26, 1.04–1.54, *p* < 0.05). M: Ns for SIT (RRR 0.78, 0.61–1.02, p > 0.05) PIT (RRR 1.20, 0.82–1.73). *Healthy*-*looking person can have HIV.* F: Ns for SIT (RRR = 1.06, 0.78–1.44). Ns for PIT (RRR 1.13, 0.88–1.46). M: *Ns* SIT (RRR 1.17, 0.72–1.89) PIT (RRR 0.77, 0.47–1.27). **Perceived future risk of HIV**. *Small risk (ref. no risk).* F: *Ns* for SIT (RRR 0.87, 0.71–1.07). Association with PIT (RRR 0.71, 0.59–0.85, *p* < 0.001). M: *Ns* SIT (RRR 0.88, 0.66–1.03) PIT (RRR 0.69, 0.51–0.92, *p* < 0.05). *Moderate risk (ref. no risk).* F: *Ns* SIT (RRR 0.83, 0.67–1.06) PIT (RRR 0.94, 0.77–1.14). M: Association with SIT (RRR 0.67, 0.51–0.89, *p* < 0.01) PIT (RRR 0.65, 0.45–0.94, *p* < 0.05). *High risk (ref. no risk).* F: *Ns* SIT (RRR 0.97, 0.72–1.31) PIT (RRR 0.91, 0.70–1.19). M: *Ns* SIT (RRR 1.15, 0.83–1.62), PIT (RRR 1.11, 0.72–1.72)

References	Location, inclusion/exclusion and testing context	Design and sampling	Measurement of psychological variables	Measure of testing behaviour	Associations between psychological variables and testing
Song et al. [63]	China Urban area MSM aged18–29 years	Cross-sectional Convenience and snowball sampling Response rate 98 % *n* = 307 Mean age 23.7 years (SD = 2.8)	*HIV-related knowledge* 20 items with dichotomous response options. Developed for current study. *α* = 0.68 *Perceived future risk for HIV* 1 item on Likert scale *Homosexuality-related stigma* 10 items on Likert scale. Developed for current study *α* = 0.93 *Willingness to test for HIV in future.* 1 item on Likert scale	Self-reported previous HIV testing	*HIV-related knowledge* *Ns* (AOR 1.04, 0.93–1.15). *Perceived future risk for HIV* Ns (AOR 0.85, 0.59–1.25). *Homosexuality-related stigma* *Ns* (AOR 1.03, 0.98–1.08). *Willingness to test for HIV in future* *Ns* (AOR 1.73, 0.87–1.58)
Stein and Nyamath [84]	U.S.A. Homeless (living in shelter 1 week or longer) Aged 15–65 years. Having a significant other willing to participate in study	Cross-sectional Response rate 90 %. *n* = 1049 428 (40.8 %) males, 621 (59.2 %) females 617 (58.8 %) African American, 176 (16.8 %) White, 243 (23.2 %) Hispanic, 13 (1.2 %) other ethnicity	*Self-esteem* 50 items with dichotomous response options. From [176] *HIV-related knowledge* 21 items with dichotomous response options. From [177] *Perceived future risk for HIV* 4 items on Likert scale. From [167] **Coping strategies in response to physical/emotional/other problems in last 6** **months** *Positive (problem*-*focused) coping* *Negative (emotion*-*focused) coping* 17 items on Likert scale. From [178]	Self-reported previous HIV testing and return for results	*Self-esteem* In women, significant correlation with testing (*r* = 0.08, *p* < 0.05). In men, *ns* (*r* = 0.01) *HIV-related knowledge* In women, significant correlation with testing (*r* = 0.20, *p* < 0.001). In men, significant correlation with testing (*r* = 0.18, *p* < 0.001) *Perceived future risk for HIV* In women, significant correlation with testing (r = 0.11, *p* < 0.05). In men, significant correlation with testing (*r* = 0.20, *p* < 0.001) **Coping strategies** *Positive (problem*-*focused) coping* In women*, s*ignificant correlation with testing (*r* = 0.19, *p* < 0.001). In men, *s*ignificant correlation with testing (*r* = 0.13, *p* < 0.05) *Negative (emotion-focused) coping* In women, *ns* (*r* = 0.06). In men, *ns* (*r* = 0.05)
Thierman et al. [85]	Zambia Urban area Provider-initiated antenatal testing Women attending antenatal clinics in selected health centres	Cross-sectional Convenience sampling Response rate >99 %. *n* = 1064 Age range 16–46 years	*Perceived risk of HIV* Developed for current study	Acceptance of antenatal HIV testing at time of study	*Perceived risk of HIV* Women with no reported risk less likely to accept testing than women reporting some risk (*p* < 0.001) Within group reporting some risk, women with low perceived risk significantly more likely to accept testing than women with moderate (*p* < 0.001) and high perceived risk (*p* < 0.001)

References	Location, inclusion/exclusion and testing context	Design and sampling	Measurement of psychological variables	Measure of testing behaviour	Associations between psychological variables and testing
Thomas et al. [86]	U.S.A. Individuals on historically black college and university (HBCU) campuses Not known to be HIV-positive Meeting age of consent for testing (in particular state)	Cross-sectional Convenience sampling. *n* = 5291. 1788 (33.8 %) males, 3499 (66.1 %) females. Age range 14–84 years (median 20 years) 5066 (95.6 %) African American, 41 (2.2 %) Hispanic, 127 (2.4 %) other ethnicity	*Perceived future risk of HIV* 1 item on Likert scale	Self-reported previous HIV testing	*Perceived future risk of HIV* High perceived risk associated with increased likelihood of testing (OR 2.00, 1.40–2.70) Medium perceived risk associated with increased likelihood of testing (OR 1.90, 1.50–2.30) Low perceived risk associated with increased likelihood of testing (OR 1.50, 1.30–1.70)
Tun et al. [64]	South Africa Urban area MSM aged ≥18 years Living in or <20 km outside Pretoria	Cross-sectional Respondent-driven sampling *n* = 307. Age range 18–42 years 288 (93.7 %) Black, 19 (6.3 %) other ethnicity	*HIV-related conspiracy beliefs* 12 items on Likert scale From [179]. *α* = 0.73 *Attitudes to condom use* 13 items on Likert scale From [150]. *α* = 0.84 *Perceived risk of HIV*	Self-reported previous HIV testing	*HIV-related conspiracy beliefs* Endorsement of conspiracy beliefs associated with never testing in adjusted (AOR 2.40, 1.10–5.70, *p* < 0.05), but not crude analysis (OR 2.20, 0.90–5.00) *Attitudes to condom use* *Ns* association between unfavourable attitudes towards condom use and never testing (OR 0.90, 0.40–2.00) *Perceived risk of HIV* *Ns* (OR 0.60, 0.20–1.50)
Wagner et al. [87]	Canada Urban area University students	Cross-sectional *n* = 770 167 (21.7 %) males, 603 (78.3 %) females Mean age 18.7 years (SD = 1.2)	*Fear of being judged negatively for HIV testing.* 32 items on Likert scale From [135]. *α* = 0.88 [135] *Social anxiety* 20 items on Likert scale. From [180]. *α* = 0.94 [180] *HIV self-relevance* Feeling of whether HIV can or will affect the participant	Self-reported previous HIV testing	*Fear of being judged negatively for HIV testing* Fear of being judged negatively by parents associated with decreased likelihood of testing (AOR 0.53, 0.33–0.87, *p* = 0.01). *Social anxiety* Social anxiety associated with decreased likelihood of testing (AOR 0.97, 0.95–1.00, *p* = 0.02) *HIV self-relevance*. Low HIV self-relevance associated with decreased likelihood of testing (AOR 1.08, 1.02–1.15, *p* = 0.02)
Wang et al. [65]	China Urban areas Rural-to-urban migrants	Cross-sectional Quota sampling *n* = 1938 1300 (67.1 %) males, 638 (32.9 %) females Mean age 25.7 years (SD = 3.5) 1880 (97 %) Han, 58 (3 %) non-Han	*Perceived peer sexual risk* 4 items on Likert scale. Developed for current study. *α* = 0.82 *Depression* 20 items on Likert scale. From [142]. *α* = 0.88 *Perceived vulnerability* Perceived vulnerability to negative consequences of risky behaviour 2 items on Likert scale. From [181] *α* = 0.80 *Perceived severity* 4 items on Likert scale. From [181], *α* = 0.60 *Satisfaction with work/life* 2 items on Likert scale. Developed for current study, *α* = 0.74 *HIV-related knowledge* 20 items with dichotomous response options. From [182]. *α* = 0.77	Self-reported previous HIV testing	*Perceived peer sexual risk* Positively associated with testing (*p* < 0.01; AOR 1.62, 1.17–2.24). *Depression* Individuals with depression more likely to have tested for HIV (*p* < 0.001) *Perceived vulnerability* Higher perceived vulnerability associated with higher likelihood of testing (*p* < 0.01). *Perceived severity* *Ns* *Satisfaction with work/life* Higher satisfaction positively associated with testing (*p* < 0.01; AOR 1.55, 1.22–1.97). *HIV-related knowledge* *Ns*
Wilkerson et al. [66]	USA Urban areas Collegiate MSM Aged 18–24 years HIV-negative	Cross-sectional Convenience sampling. *n* = 930. Mean age 20.7 years. 653 (72.2 %) White, 29 (3.2 %) Black, 133 (14.7 %) Hispanic, 90 (9.9 %) Other	*Internalised homonegativity* 7 items on Likert scale From [166]. *α* = 0.74 *Openness of homosexuality* 1 item on Likert scale *Community acceptance of homosexuality* 7 items on Likert scale *α* = 0.69	Self-reported annual HIV testing	*Internalised homonegativity* *Ns* association with annual testing uptake (AOR 1.00, 0.80–1.20) *Openness of homosexuality* Significant association with annual testing uptake (AOR 1.30, 1.10–1.50, *p* < 0.05) *Community acceptance of homosexuality* *Ns* association with annual testing uptake (AOR 0.90, 0.70–1.20)
Yi et al. [88]	Cambodia VCT. Tuberculosis patients attending selected hospitals. Aged 15–49 years	Cross-sectional Response rate 98.9 %. *n* = 154 75 (49 %) males, 79 (51 %) females Mean age 34.6 years (SD = 7.9)	*HIV-related stigma* 13 items with dichotomous response options, From [77]	Self-reported previous uptake of VCT	*HIV-related stigma* *‘PLWHA are dirty’—*associated with never testing (OR 2.30, 1.04–5.40) *‘PLWHA must have done something wrong’—*associated with never testing (OR 4.2, 1.65–11.11) *‘I* *would be uncomfortable with a neighbour who has AIDS’—*associated with never testing (OR 3.00, 1.26–7.42)

### Participants

Across all studies, there were 339,227 participants. Sample sizes were generally large (the largest sample size was 134,965 [38] ) and 28 studies had sample sizes of over 1,000 [36–39, 41, 44, 50, 51, 53, 56, 58, 60–62, 65, 68, 70, 71, 75, 78, 81–86, 90, 91]. Only one study [40] had a sample size below 100. There was a diverse range of target populations. Most studies had wide age ranges, with participants aged 15–60 years. Exceptions included one study that sampled high school students [70], seven that sampled university students [57, 61, 80, 86, 87, 89, 92], and two studies that sampled adults aged 50 and older [42, 91]. Other studies sampled populations at higher risk for HIV: two studies sampled intravenous drugs users (IDU) [40, 78], five sampled sexually transmitted infection (STI) clinic attendees [11, 52, 59, 67, 73, 74] sampled men who have sex with men (MSM) [31, 41, 43, 45, 46, 49, 55, 60, 63, 64, 66], two sampled female sex workers (FSW) [44, 51], and one sampled male clients of FSW [79]. One study sampled patients receiving care for tuberculosis [88], and two sampled women attending antenatal care [58, 85]. One study [78] included several high-risk groups in its analysis (IDU, MSM, heterosexual individuals recruited from gay bars, and STI clinic attendees). Two studies sampled inmates of correctional facilities [33, 69]. Gender ratios varied between studies, but there was an overall majority of male participants (approximately 55 %).

Twenty-eight studies reported the ethnicity of participants [32, 35, 40, 44–47, 49, 53, 55, 57, 59, 61, 64–67, 71–74, 77, 78, 80, 84, 86, 90, 92]. At least eight different ethnic groups were represented (African American, Black African, White, Asian/Pacific Islander, Hispanic, Han Chinese, Non-Han Chinese and Native American).

### Measurement of Testing Behaviour

Of the 56 cross-sectional studies, 49 (88 %) used self-report measures to assess testing [31, 34–44, 46, 49–53, 56, 57, 59–70, 72, 73, 75–80, 82–84, 86–88, 90, 91], with participants reporting whether they had tested for HIV. In the majority of studies (n = 34) [34, 36, 37, 39, 44, 46, 50, 51, 53, 56, 57, 59, 61–65, 67, 68, 70, 72, 73, 75–78, 80, 82, 83, 86–88, 90, 91], participants were asked to specify whether they had ‘ever’ been tested for HIV. Five studies asked participants to specify whether they had tested in the last 12 months or previously in their lifetimes [35, 41, 43, 49, 60]. Three studies asked participants if they had tested in the last 12 months [42, 48, 69], and two asked participants if they had tested in the last 6 months [52, 79]. Two studies asked participants if they had both been tested and returned for results [38, 84]. Three studies measured frequency of testing, either by summing the number of times participants had tested [40] or categorising testing as either ‘routine/non-routine’ or annual [31, 66].

Twelve studies assessed testing behaviour either at the time of study or during a specified follow-up period. In general these relied on clinical records, such as blood draws [32, 47, 81] or medical logs [33, 58, 74, 85], to establish testing behaviour. Exceptions included three prospective cohort studies [45, 55, 71] and one intervention study [48], which used self-report measures to assess whether participants had tested during follow-up, and one cross sectional study, which measured self-reported testing uptake at the time of the study [92].

### Measurement of Psychological Factors

A number of studies used health behaviour theories to direct the measurement psychological variables, most commonly the Health Belief Model [32, 51, 80, 92]. There was considerable variation in the type of psychological variables measured across studies. These were grouped into variables specifically related to testing (e.g. perceived benefits and barriers to testing), HIV non-testing variables (e.g., HIV-related stigma, and HIV-related knowledge), sexual behaviour cognitions (e.g., peer sexual norms and attitudes towards condom use), general psychological variables (e.g., depression, self-esteem) and societal cognitions (e.g., perceived social support, institutional mistrust, and homosexuality-related stigma). Perceived HIV risk was the most commonly measured variable, in 28 studies [33, 40, 42, 44, 46–48, 52–54, 56, 57, 62–64, 69, 73, 79, 81–86, 89, 91, 94, 95]. HIV-related knowledge was measured in 25 studies [31, 33–35, 39, 42–44, 46, 48–50, 52, 56, 58, 61–63, 65, 67, 73, 77, 79, 83, 84]. Eighteen studies measured HIV-related stigma [31, 33, 34, 36, 38, 40, 41, 50, 51, 59, 62, 67, 75–77, 87, 90, 96].

### Relationships Between Psychological Variables and Testing

Meta-analyses were carried out on the relationship between HIV testing and the variables of HIV-related knowledge and perceived risk of HIV, given the larger number of studies measuring these variables where data was available (>15 studies). Findings will be discussed in relation to individual psychological variables where these appeared in two or more studies.

#### HIV Testing-Related Psychosocial Variables

##### Perceived Benefits of Testing/Pro-testing Attitudes

The majority of studies showed positive relationships between perceived benefits of testing and testing behaviour. Of eight studies, six found a significant positive relationship with testing (previous testing or test acceptance on the same day). These six studies sampled from varied populations, two [31, 41] were conducted with MSM, two [92, 97] with university students, one with prisoners [69] and one [77] with residents of a peri-urban setting in South Africa. One study [32] that found a non-significant relationship between perceived benefits and testing measured test acceptance on the same day (with women who had experienced intimate partner violence). One study [51] found generally non-significant relationships between perceived benefits and testing, although men on worksites and low income women tested *less* if they perceived testing to be useful in HIV-negative individuals. Only two of these eight studies took place in sub-Saharan Africa [51, 77].

##### Perceived Barriers to Testing/Cons of Testing

Five of the eight studies which measured perceived barriers to testing found an association with testing in either univariate or multivariate analysis (lower perceived barriers significantly associated with previous testing) [31, 51, 57, 76, 80]. Five of the eight studies took place in resource rich contexts [31, 32, 57, 80, 92]. Studies assessed a range of barriers including uncertainty about confidentiality, fear of needles and perceived difficulty in obtaining an HIV test.

##### Perceived Accessibility and Knowledge of Testing Site

‘Knowledge of a testing site/services’ or perceived accessibility of testing site was measured (using a single item) in four studies [46, 52, 60, 76]. All four found highly significant positive relationships with previous testing with three of the four studies showing independent effects [46, 60, 76]. These studies took place in a variety of settings and with different populations.

##### Perceived Behavioural Control/Self-efficacy

Perceived behavioural control in relation to testing includes both internal and external control factors. Two studies [31, 43] (both with MSM) measured perceived behavioural control and found significant independent associations with previous testing. One study found a large independent effect of the related construct of testing self-efficacy on testing [98].

##### Perceived Norms of Testing

There were inconsistent relationships between perceived testing norms and testing. Four studies measured descriptive norms (beliefs about the testing attitudes and behaviour of others). Two studies found significant independent positive relationship between descriptive norms and previous testing, using single items [41, 59]. Two studies, however, failed to find relationships between descriptive norms and testing [75, 92]. One study [31] measured subjective/social norms (perceived social pressure to test). They found, in an MSM sample, a significant positive relationship between subjective norms (belief that friends would endorse the participant’s decision to test for HIV) and previous testing in univariate but not multivariate analysis.

##### Fear of Testing

Three studies [31, 77, 78] measured fear of testing. All three found significant negative associations with previous testing, although not in multivariate analysis in one study [31].

##### Intention to Test in the Future

Studies generally supported a positive relationship between intention to test, and testing behaviour. Four studies measured intention to test for HIV in the future. Three [35, 43, 58], observed an effect on testing, although one study only found a univariate and not a multivariate effect [35]. One of these was a prospective cohort study [58] with women attending antenatal care, the other two [35, 43] measured testing behaviour retrospectively. The fourth study [76], carried out with Tanzanian school teachers, showed a non-significant relationship between intention and testing.

#### Non Testing HIV-Related Psychosocial Variables

##### HIV-Related Knowledge

**Fig. 2:**
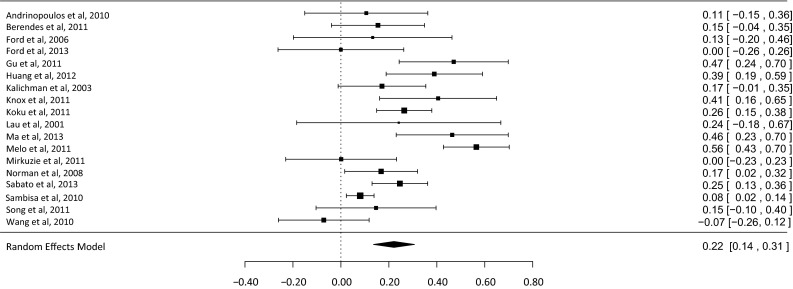
Effect sizes for HIV-related knowledge and HIV testing (*d*)

Of the 25 studies measuring HIV-related knowledge, 14 found a significant positive association with testing [31, 34, 35, 39, 43, 46, 49, 50, 52, 56, 58, 61, 62, 84]. One [61] found a significant association among female but not male participants. A random effects meta-analyses found a small [99] positive association between HIV-related knowledge and lifetime testing (*d* = 0.22, 95 % CI 0.14–0.31, *p* < 0.001). A similar level of significance was found using permutation testing (*p* = 0.002). Significant heterogeneity was found across studies (*I*^*2*^ = 77.28 %, *Q* = 75.75, *p* < 0.001, see Fig. 2).

The association between HIV knowledge and testing was not moderated by high income versus low/middle income study setting (*p* < 0.46). One outlier [56] was identified from the meta-analysis. Removal of this study from the model resulted in minimal change (*d* = 0.20, 95 % CI 0.12–0.27, *p* < 0.001). There was little evidence of publication bias (Rosenberg’s Fail-Safe N = 479), with the trim and fill method estimating only one missing study was contributing to funnel plot asymmetry.

##### Perceived Risk of HIV

A distinction was made between studies measuring participants’ perceived risk of currently being HIV-positive (n = 3) [33, 46, 47], participants’ perceived risk of acquiring HIV in the future (n = 15) [40, 42, 44, 51, 62, 63, 69, 72, 79, 82–84, 86, 91, 100], and studies where it was unclear if the measure referred to current or future risk (n = 10) [48, 52–54, 56, 57, 64, 73, 81, 85]. Of three studies measuring participants’ perceived risk of currently being HIV-positive, one study [33] found a significant positive association with testing and two did not [46, 47]. Of the 15 studies measuring participants’ perceived risk of contracting HIV in the future, eight found significant positive relationships with testing [40, 62, 72, 82–84, 86, 91], one of these only in women and not in men [72], and one more frequently for provider-initiated than client-initiated testing [62]. One study [51] found a significant *negative* association between perceived risk and testing (among female sex workers only). Of the ten studies that did not specify whether they were measuring either present/future perceived risk, four found a significant positive association with testing [52, 53, 56, 57]. Two [52, 53] of these found significant associations among male, but not female participants.

**Fig. 3:**
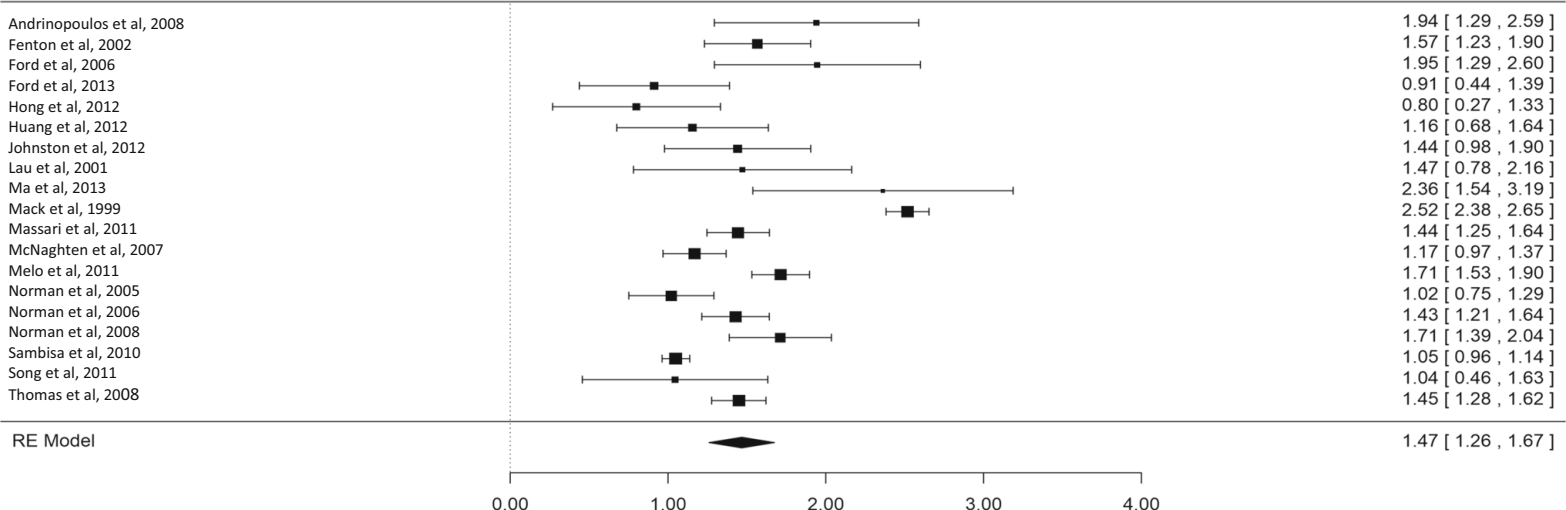
Effect sizes for HIV risk perception and HIV testing (ORs)

Due to the relatively small number of studies for each of the risk variables and the conceptual similarity in measurement, all measures of perceived risk (current/future/unknown) were included in the same meta-analysis. A small positive association was found between perceived risk of HIV and lifetime testing using a random effects meta-analysis model (OR 1.47, 95 % CI 1.26–1.67, *p* < 0.001). A similar level of significance was found using permutation testing (*p* = 0.002). There was significant heterogeneity across studies (*I*^*2*^ = 92.01 %, *Q* = 369.07, *p* < 0.001, see Fig. 3).

The association between risk perception and HIV testing was not moderated by high income versus low/middle income study setting (*p* = 0.19). One outlier [91] was identified from the meta-analysis. Its removal did not significantly affect the model (OR 1.38, 95 % CI 1.23–1.53, *p* < 0.001). There was no evidence of publication bias (Rosenberg’s Fail-Safe N = 15,207), with the trim and fill method estimating zero studies were missing from the left side of the funnel plot.

##### HIV-Related Stigma

Earnshaw and Chaudoir’s HIV stigma framework [101] was used to categorise the different measures of stigma used.

*Prejudiced attitudes* Ten studies measured prejudicial attitudes towards people living with HIV (PLWH) [34, 36, 38, 40, 50, 51, 59, 62, 77, 88]. Five studies found that holding prejudicial attitudes was significantly associated with lower uptake of previous testing [38, 50, 59, 77, 88]. A further two studies found some associations between attitudes towards PLWH and HIV testing [34, 36]. The studies measuring prejudiced attitudes covered a variety of populations and contexts.

*Discrimination* Discrimination against PLWH was measured in four studies [40, 59, 62, 90]. One of these studies [62], using data from a population-based survey in Zimbabwe, found a significant negative association (for both client and provider-initiated testing) among female, but not male participants. The other three studies failed to show an effect [40, 59, 90].

*Anticipated stigma* Anticipated stigma if diagnosed HIV-positive or testing for HIV was measured in three studies. Two studies failed to show an effect with testing [33, 62]. One study found that anticipated stigma was associated with an absence of testing in univariate but not multivariate analysis [76].

*Mixed measures of stigma* There were two studies where the stigma measures could not be categorised according to the Stigma Framework [101] (due to the use of scales which combined items from across categories). One study found that stigma was associated with an absence of testing in univariate but not multivariate analysis [31]. The second study found that stigma was associated with lower levels of testing in Thailand but not in African sites [75].

Meta-analysis was not carried out on the relationship between HIV stigma and HIV testing due to the small number of studies measuring each distinct stigma process.

##### Perceived Susceptibility to HIV

There was inconsistent evidence on the relationship between perceived susceptibility and testing. Of seven studies measuring perceived susceptibility to HIV, two [32, 80] found a significant positive association with testing. One study [37] found higher perceived susceptibility was significantly associated with *less* likelihood of previous testing. The four studies with non-significant findings [31, 45, 76, 92] assessed a variety of populations including MSM, college students, and school teachers.

##### Perceived Severity of HIV

There was no evidence supporting a relationship between perceived severity of HIV and testing. Of the three studies [31, 32, 92] measuring perceived severity of HIV, none found a significant relationship with testing.

##### Fear of HIV Infection

Two studies [41, 43] measured fear of contracting HIV. Both found increased fear of HIV was independently significantly associated with decreased likelihood of testing. Both studies were conducted with MSM.

##### Belief in HIV-Related Conspiracy Theories

There was contradictory evidence on the direction of the effect for belief in conspiracy theories and testing. Four studies measured belief in HIV-related conspiracy theories. Two studies [42, 68] found that holding conspiracy beliefs was associated with a greater likelihood of testing. Two studies [64, 67] found significant negative associations with testing.

##### Knowing Someone with HIV

Of eight studies which asked whether participants knew someone with HIV (two studies [70, 83] specifically asking if the participant had a friend or relative with HIV), six [69, 70, 82, 83, 90, 100] reported a significant independent positive relationship between knowing someone with HIV and testing. These studies took place in different contexts and with different populations.

#### Sexual Behaviour Cognitions

##### Peer Sexual Norms

One study [65] measuring perceived peer sexual risk-taking, found a significant positive association with previous testing. One study [72] measuring descriptive norms of using condoms with new partners, found that lower perceived norms was associated with less likelihood of previous testing.

##### Attitudes to Condom Use

Neither of the two studies [64, 79] measuring attitudes towards condom use found a significant relationship with testing.

##### Sexual Self-efficacy/Sexual Locus of Control

Two studies [34, 37] measured self-efficacy for HIV preventative behaviours, in African populations. Both found a significant positive relationship with previous testing using multi-item scales. One study [61] in the US measuring participants’ locus of control for sexual activities found that greater internal control was associated with a higher likelihood of testing.

#### General Psychological Variables

##### Depression

There was conflicting evidence on the effect of depression on testing. Of three studies measuring depression [61, 65, 68] one [68] found a significant negative association, and one [65] found a significant positive association with previous testing.

##### Coping Mechanisms

Two studies [74, 84] measured coping mechanisms in response to stressors. One study found that problem-focused/positive coping strategies were positively associated with testing [84]. The second study [74] did not find any relationship between coping and testing.

##### Self-efficacy for Handling Difficult Situations

Of two studies [32, 48] measuring self-efficacy for the general handling of difficult situations, neither found a significant relationship with testing.

##### Perceived Health Status

Of the two studies which measured the self-perceived health of the participants, one study in Tanzania [76] found that those with more positively-rated health status had a higher likelihood of testing. The other in Eastern Europe [70] found that participants with more poorly rated health status had a higher likelihood of previous testing.

#### Societal Cognitions

##### Perceived Social Support

Of the two studies [33, 53] measuring perceived social support, neither found a significant relationship with testing.

##### Institutional Mistrust/Perceived Discrimination

Three studies measured different aspects of institutional mistrust. Two found a significant negative association between previous testing and beliefs in systematic discrimination [45], and government mistrust [42]. One study [74] found a *positive* association between perceived racism and testing.

##### Homosexuality-Related Stigma

Three studies measured internalised homophobia. One [49] found a significant negative association with previous testing, two failed to show an effect [66, 98]. One study [66] also measured openness of homosexuality and found a significant positive association with previous testing. Sexual orientation-based discrimination/stigma was measured by four studies [43, 49, 63, 66]. Only one study showed a relationship between discrimination and testing [43].

### Methodological Quality

The methodological quality of studies is summarised in Table 3. A tick (✔) signifies that the criterion was met. A cross (x) indicates that the criterion was either not met or it was unclear if the criterion was met.

**Table 3:** Methodological quality ratings

References	External validity		Internal validity			
	Representativeness of sample	Percentage of selected individuals who agreed to participate	Measurement of testing behaviour	Measurement of psychological factors	Attrition rate: percentage of participants included in final analysis	How far confounding variables are measured/analysed appropriately
Adam et al. [31, 41]	×	×	×	✔	–	✔
Andrinopoulos et al. [33]	✔	✔	✔	✔	–	✔
Berendes and Rimal [34]	✔	×	×	×	–	×
Berkley-Patton et al. [35]	×	×	×	✔	–	✔
Bogart et al. [67]	×	×	×	✔	–	✔
Bohnert and Latkin [68]	×	×	×	✔	–	✔
Burchell et al. [69]	✔	✔	×	×	–	✔
Corno and de Walque [36]	✔	×	×	✔	–	✔
Creel and Rimal [37]	✔	×	×	✔	–	✔
Cremin et al. [38]	✔	✔	×	×	–	✔
Das et al. [39]	✔	✔	×	✔	–	✔
Delva et al. [70]	✔	×	×	×	–	✔
Desai et al. [71]	×	×	×	✔	✔	✔
Dorr et al. [92]	×	×	✔	✔	–	✔
Earnshaw and Chaudoir [101]	×	×	×	✔	–	×
Fenton et al. [72]	×	×	×	×	–	✔
Flowers et al. [41]	✔	×	×	×		✔
Ford et al. [73]	×	✔	×	✔	–	×
Ford et al. [74]	×	✔	✔	✔	–	✔
Ford et al. [42]	✔	×	×	✔	–	✔
Gu et al. [43]	×	✔	×	×	–	✔
Hendriksen et al. [75]	✔	×	×	✔	–	×
Hong et al. [44]	✔	×	×	×	–	✔
Hoyt [45]	×	×	×	✔	×	✔
Huang et al. [46]	×	×	×	✔	–	✔
Johnston et al. [47]	×	×	✔	×	–	✔
Kakoko et al. [76]	×	✔	×	✔	–	✔
Kalichman and Simbayi [77]	×	×	×	✔	–	✔
Kaufman et al. [115]	✔	×	×	✔	×	✔
Kellerman et al. [78]	×	×	×	×	–	×
Knox et al. [49]	×	×	×	✔	–	✔
Koku [50]	✔	×	×	×	–	✔
Lau and Wong [79]	×	×	×	×	–	×
Lofquist [51]	✔	✔	×	×	–	✔
Ma et al. [52]	×	×	×	✔	–	×
Mack and Bland [91]	✔	×	×	×	–	✔
MacPhail[90]	✔	×	×	×	–	✔
Maguen et al. [80]	×	×	×	✔	–	✔
Massari et al. [53]	✔	×	×	✔	–	✔
Matovu et al. [54]	✔	×	×	×	–	✔
McGarrity and Huebner [55]	×	×	×	×	–	×
McNaghten et al. [81]	✔	×	✔	×	–	×
Melo et al. [93]	✔	✔	×	✔	–	✔
Menser [97]	×	×	×	✔	–	×
Mirkuzie et al. [58]	×	✔	✔	×	✔	✔
Norman and Gebre [89]	×	×	×	×	–	✔
Norman, [82]	✔	×	×	×	–	✔
Norman et al. [83]	×	×	×	×	–	✔
Pettifor et al. [59]	×	×	×	✔	–	✔
Prati [60]	×	×	×	×	–	✔
Ratcliff et al. [32]	×	×	✔	✔	–	✔
Sabato et al. [61]	×	×	×	✔	–	✔
Sambisa et al. [62]	✔	✔	×	×	–	✔
Song et al. [63]	×	✔	×	✔	–	✔
Stein and Nyamathi [84]	×	✔	×	✔	–	×
Thierman et al. [85]	×	✔	✔	×	–	×
Thomas et al. [86]	×	×	×	×	–	×
Tun et al. [64]	×	×	×	✔	–	✔
Wagner et al. [87]	×	×	×	✔	–	✔
Wang et al. [65]	×	×	×	✔	–	✔
Wilkerson et al. [66]	×	×	×	✔	–	✔
Yi et al. [88]	×	✔	×	✔	–	×

#### External Validity

Twenty-three of the 62 studies used random sampling [33, 34, 36–39, 41, 42, 44, 48, 50, 51, 53, 54, 56, 62, 69, 70, 75, 81, 82, 90, 91], and 33 used consecutive sampling methods [31, 32, 35, 40, 43, 45–47, 49, 52, 55, 57–60, 63–68, 72–74, 76–78, 80, 83, 85, 86, 92, 100] (see Table 3). Six studies did not specify the sampling method used [61, 71, 79, 84, 87, 88]. Twenty-three studies reported response rates [31, 33, 38–41, 43, 44, 51–53, 56, 58, 62, 63, 69, 72–74, 76, 84, 85, 88], with 16 studies specifying that at least 80 % of those eligible to participate were recruited [33, 38, 39, 43, 51, 56, 58, 62, 63, 69, 73, 74, 76, 84, 85, 88]. Only seven studies met both criteria for external validity [33, 38, 39, 51, 56, 62, 69].

#### Internal Validity

Eight studies measured testing objectively, using the provision of a blood specimen at the time of study, or clinic records [32, 33, 47, 58, 74, 81, 85, 92]. Thirty-five studies measured psychological variables using methods of established reliability and validity [31–33, 35–37, 39, 40, 42, 45, 46, 48, 49, 52, 53, 56, 57, 59, 61, 63–68, 71, 73–77, 80, 84, 87, 92]. Two of the four prospective cohort studies [58, 71] were free from attrition bias, reporting that at least 80 % of participants were present in the final analysis. One study [45] did not provide enough information for attrition rate to be established. One prospective cohort study [55] and the intervention study [48] reported attrition rates of over 20 %. Forty-nine studies carried out multivariate analyses to control for potential confounding variables [31–33, 35–39, 41–51, 53, 54, 56, 58–72, 74, 76, 77, 80, 82, 83, 87, 90–92, 100]. In total, only four of the 62 studies provided evidence of meeting all criteria for internal validity [32, 33, 74, 92].

## Discussion

This review aimed to synthesise and analyse data from studies investigating the relationship between psychological variables and HIV testing. Sixty-two studies were included. The most commonly measured variables were either directly related to HIV testing (e.g., perceived benefits of and barriers to testing) or HIV non-testing related variables (e.g., HIV knowledge). In general, there appeared to be larger effects for proximal testing-related variables (e.g., HIV testing fear) than for more distal variables (e.g., depression). The generally large sample sizes suggest that a lack of statistical power is an unlikely explanation for many of the small effects reported.

Many HIV-testing related variables included in studies are featured in health behaviour models [102–104]. Perceived benefits of testing were associated with HIV testing in the majority of studies which assessed this variable, with strong independent relationships across different populations and contexts [31, 69, 77, 92]. There were inconsistent findings, however, of the effects of perceived barriers or cons of testing. Assessing the effect of this variable on testing is complex partly because it has been measured as both a multi-dimensional construct [51] and as its individual components (e.g., testing fear, anticipated stigma, perceived accessibility of testing). Perceived behavioural control or testing self-efficacy were infrequently measured. All three studies that measured these variables found significant positive relationships with testing [31, 43, 60]. There were mixed findings in relation to normative beliefs. Descriptive norms (beliefs about the testing attitudes and behaviour of others) were more frequently measured than subjective norms (perceived social pressure to test). This is despite the fact that descriptive norms do not appear in the most commonly used health behaviour models, in contrast to subjective norms [102]. Intention to test in the future was only independently associated with HIV testing in two of four studies [43, 58]. It is likely that a number of other factors, including some of those reported in this review, are associated with the likelihood of intention being enacted. For all of the above constructs, very few studies were conducted in sub-Saharan Africa and the majority used scales with five items or fewer.

Fear of testing was significantly associated with testing in all three studies, in different populations, where this was assessed [31, 77, 78]. Fear of HIV infection also showed negative relationships with HIV testing (in two studies) consistent with the effect of fear of HIV testing [41, 43]. These findings are in contrast to the lack of an effect of perceived severity of HIV despite the latter factor appearing in some health behaviour models [104, 105]. It may be that other aspects beyond HIV severity contribute to fear responses. Emotional factors are rarely directly included in health behaviour models with some exceptions [106, 107]. The small number of studies where fear was measured may underplay its significance in the HIV testing context. The fear findings are consistent with conceptualising HIV testing as a detection behaviour associated with significant personal risks. Prospect Theory [108] states that people are fundamentally risk averse and in certain situations (perhaps when the outcome of the behaviour is uncertain) people will choose not to act rather than face the risk of a negative outcome if they engage in the target behaviour (e.g., testing positive for HIV as a result of taking an HIV test).

Small positive associations between perceived HIV risk and HIV testing (and between HIV knowledge and HIV testing) across different populations and contexts were found, consistent with potential distal effects. The relationship between perceived HIV risk and HIV testing is difficult to interpret given measurement ambiguity. In some studies, HIV risk referred to beliefs about currently being HIV positive. More commonly, HIV risk referred to an estimation of the likelihood of becoming HIV positive in the future (very similar to perceived susceptibility). In many studies, it was unclear whether the measure referred to current or future risk perception or whether the authors intended to distinguish the variable from perceived susceptibility. It may be that there are different relationships between current HIV risk and testing and future HIV risk (or susceptibility) and HIV testing. Many models of health behaviour include the construct of HIV risk perception or susceptibility [13, 105, 109, 110], with the effect of risk perception or perceived health threat sometimes thought to be mediated by appraisal and coping processes [106].

HIV-related stigma was measured in many studies (using multi-item scales), despite its lack of inclusion in the most commonly used health behaviour models. We used an HIV stigma framework [101] to organise findings but it remained difficult to clarify the intended nature of many measures. The strongest effect appeared to be a negative relationship between prejudiced attitudes towards PLWH and HIV testing. Other aspects of HIV stigma (discrimination against PWLH and anticipated stigma) or mixed measures of stigma appeared to be less strongly related to HIV testing.

There was an effect of knowing someone with HIV on testing. If the known person with HIV was a sexual partner, this may have triggered HIV testing, consistent with the impact of social messages on illness representation [106] or as a cue to action [104]. As studies tended to ask a single question to assess this variable, it was not possible to ascertain whether the identity of the known person had an effect on testing. In addition, given the historical nature of the outcome variable in many studies, the direction of possible causation is unclear.

The relationship between higher levels of sexual self-efficacy/sexual locus of control and greater rates of HIV testing in all three studies where this was measured [34, 37, 61] was surprising. This factor does not appear in health behaviour models. It may be that this aspect of self-efficacy is conceptually related to HIV testing self-efficacy/perceived behavioural control, which has been invoked in health behaviour models.

### Strengths and Limitations of the Review

One of the main strengths of the review was its broad inclusion criteria. This was reflected in a comprehensive search strategy which included peer-reviewed journals and grey literature, with no regional and few population restrictions. The wide range of participant characteristics in the included studies enhances external validity and potentially allows one to assess whether these characteristics moderate the relationship between psychological factors and HIV testing. The use of meta-analysis in this context is novel, as is the use of permutation tests [111] to corroborate the findings from random effects models, given the relatively small number of studies included. Some moderator analysis was conducted, although there was only sufficient data available to examine one moderator (country income level) on the relationships between risk perception and testing, and HIV knowledge and testing. It will be important for future studies to be able to determine whether the relationship between a wider range of psychological variables and HIV is moderated by study location. For example, there may be differences in whether fear about testing influences testing uptake in different contexts.

It was not possible to carry out meta-analysis on a wider range of variables. Therefore, it cannot be concluded that those variables where the majority of studies show a significant relationship with testing equates with pooled estimates that show significant testing effects. As more studies are carried out, researchers will be able to carry out such analysis as well as moderation analysis of significant power to be able to detect significant effects for a range of potential moderators (e.g., sex, provider versus initiator testing, sexual preference) [112]. We used multiple methods of assessing potential publication bias, although we acknowledge limitations with existing techniques [113]. A further limitation of the review related to the grouping of independent variables. There was considerable variation in measures and terminology used. The Theoretical Domains Framework was considered as a tool to organise independent variables but this was rejected as the Framework appeared to be at too high a level of abstraction to capture the complexity of measures used [114]. Inevitably, with many overlapping constructs and with some measures of uncertain reliability and validity, this may have influenced the nature and magnitude of summarised effects. In particular, it may be that combining risk perception measures in the same meta-analysis may have obscured the effects of current versus future risk perception. This review did not examine relationships between models in their entirety and testing, although the findings on individual variables suggest that current models might require modifications for them to be applied validly to HIV testing contexts.

## Research Implications

An important limitation of studies that aimed to answer questions about associations between psychological factors and HIV testing was the retrospective measurement of HIV testing. Examining the relationship between current psychological variables and lifetime HIV testing complicates casual inferences. For example, it may be that people’s perceptions of their risk of HIV (current or future), or their perceived benefits of HIV testing are post hoc rationalisations of the outcome of previous HIV testing. It would be helpful for more studies to use prospective designs to examine relationships between psychosocial variables and HIV testing. Only one intervention study [115] was included in this review as, typically, testing interventions did not measure associations between potentially mediating psychological variables and testing. Doing so would be helpful to establish the causal mechanism of interventions. It would also be useful for studies to clarify whether testing took place as a result of a client or provider-initiated process.

Most studies measured cognitions in contrast to assessing emotions. It would be useful to see a greater emphasis on measuring emotions (e.g., anxiety and guilt), particularly given the associations seen between fear and HIV testing. Regarding variables that were measured, we suggest that testing benefits and barriers, perceived behavioural control (along with other aspects of self-efficacy such as sexual self-efficacy), and normative beliefs be included more frequently in future studies. We argue that using multi-item scales to measure these constructs [116, 117] are likely to be more reliable and valid that the briefer scales that are more commonly used. We also suggest that such work be carried out in sub-Saharan Africa, given the limited research on these factors in this context. Both current and future risk perception could be assessed in the same study in the future and they should be distinguished and clearly defined. In addition, it would also be useful to ask separately about individuals whom participants know are HIV-positive.

## Practice Implications

This review did not directly assess interventions to increase HIV testing and, in general, interventions have not assessed their effects on mediating psychological variables. Hence, any practice implications must be expressed cautiously. At the most, we can only suggest variables that could be both be targeted in interventions and measured as potential mediators of the effects of interventions on HIV testing.

On the basis of the evidence in this review, it would seem fruitful to focus on interventions that emphasise the benefits of testing, enhance testing self-efficacy, provide information on testing sites, minimise HIV testing fear, decrease prejudice towards PLWH and increase personal contact with PLWH. Interventions targeting these factors can be delivered at a range of levels. That is, change at higher levels could facilitate change in proximal psychological determinants of testing. At the individual level, approaches such as motivational interviewing (with the aim of supporting self-efficacy and building on the individual’s perceived benefits for testing) have been used with some success [118–120]. At the social/relational level, peer education may also help to change testing attitudes and self-efficacy as well as providing information on testing availability. Peer education has been used successfully to enhance HIV testing rates [121]. At the population level, mass media and social marketing approaches may influence similar testing determinants. Both have been used with some evidence of enhanced HIV testing rates [122–124]. Finally, structural approaches to increase the availability, acceptability and accessibility of HIV testing, may influence intrapersonal psychological factors. There is considerable evidence of the effectiveness of structural approaches such as rapid, provider-initiated, mobile and home testing in enhancing HIV testing rates [125–128].
